# Global Genetic Diversity, Provisional Strain Demarcation, and Evolutionary Dynamics of Potato Virus X (PVX)

**DOI:** 10.3390/v18090968

**Published:** 2026-09-02

**Authors:** Farah Naz, Muhammad Tahir

**Affiliations:** Department of Agricultural Sciences & Technology (AST), Atta-ur-Rahman School of Applied Biosciences (ASAB), National University of Sciences and Technology (NUST), H-12, Islamabad 44000, Pakistan

**Keywords:** potato, potato virus x, genetic diversity, evolution, recombination, geographical distribution, host range

## Abstract

Potato virus X (PVX) is an economically important pathogen of potato (*Solanum tuberosum*), causing global yield losses of 30–40% annually in single infections and up to 80% in mixed infections. To analyze genetic diversity and evolution, 413 full-length PVX isolates available in the databases were investigated. Pairwise nucleotide sequence identities using the Sequence Demarcation Tool (SDT) suggested a tentative, dataset-dependent species and strain demarcation threshold of 76% and 89%, respectively, delineating eleven (11) putative strains, named alphabetically from A to K. Phylogenetic analysis strongly supported the provisional strain demarcation, showing distinct and non-overlapping phylogenetic clusters. PVX-A constitutes a phylogenetically heterogeneous lineage, potentially contributing to its evolutionary success and long-term persistence. The PVX population exhibited high nucleotide and haplotype diversity, elevated gene flow and a moderate level of genetic differentiation among the geographically distinct clusters. Recombination analysis, using RDP, identified 20 recombination events involving 50 recombinant isolates, while the GARD program detected only a single breakpoint at position 6229 bp within the coat protein (CP) region. Recombination, alongside the nucleotide substitution rate and gene flow, was identified as a secondary evolutionary force driving PVX diversity. The Andean region was identified as the probable origin of PVX, from which it spread to Europe through early transcontinental human activities and subsequently disseminated across the world. The Andes and Europe likely represent two major evolutionary niches. PVX has a moderate host range, with potato as its prime natural host, and the majority of PVX genotypes infect only potato. Among all PVX genomic lineages, PVX-A shows the widest geographical distribution and host range, reported in almost every potato-growing country, including Pakistan. This study provides a comprehensive framework for understanding PVX diversity, provisional strain demarcation, evolution, and global dispersal, facilitating acquiring sustainable resistance and improving preventive strategies against PVX for global potato cultivation.

## 1. Introduction

Agriculture is one of the world’s most vital sectors, accounting for approximately 4% of global GDP and responsible for the food security of almost 8 billion people [1,2,3,4,5,6]. Potato (*Solanum tuberosum* L.) ranks as the third most important food crop globally, after wheat and rice, in terms of consumption [2,7]. Cultivated across 150 countries [8,9], the potato has over 4000 edible varieties, with the majority found in the Andes region [10,11]. Potato is a rich source of carbohydrates (energy), starch, high-quality proteins, dietary fibers, lipids, vitamins, and minerals [12,13,14,15]. Furthermore, the tuber possesses anti-carcinogenic, anti-inflammatory, and anti-hypertensive properties, which promote human health [9,14,15,16,17].

**Figure 1:**
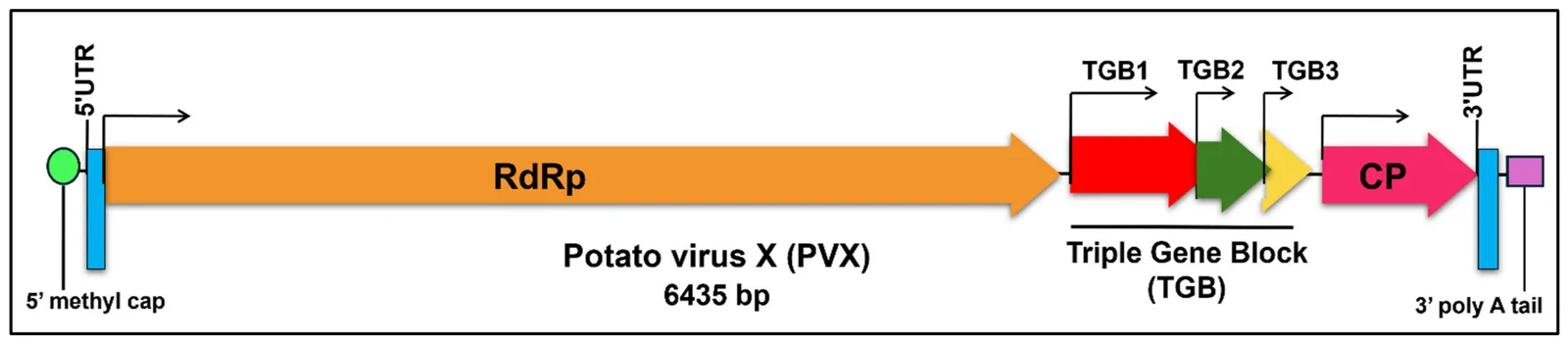
Complete genome of potato virus X with nucleotide length of 6435 bp. Boxes at both ends of the genome represent the 5′ methyl cap, UTRs, and 3′poly A tail. The first arrow indicates the longest ORF 1 of the genome, encoding RdRp, which is involved in replication. The next three arrows represent overlapping ORFs 2, 3, and 4 (TGB1, TGB2, TGB3, collectively called triple gene block) involved in virus movement. An arrow near the 3′ end shows ORF 5, which encodes CP, which plays a role in encapsidation of virion particles.

One of the major challenges to potato yield and production is potato viruses. Over 50 viral species are known to infect potato crops worldwide [7,9,18]. Among these, the most economically damaging include potato virus X (PVX), potato virus Y (PVY), and potato leaf roll virus (PLRV) [18,19,20,21,22]. Potato virus X holds historical significance as one of the earliest identified potato viruses, having been first reported in the United Kingdom by Smith in 1931 [22,23,24,25]. Potato virus X (PVX) belongs to the genus Potexvirus, which is a populous member of the family Alphaflexiviridae, comprising 52 species. In infected host plants, PVX typically induces symptoms such as mild mosaic, inter-veinal chlorosis, apical necrotic spots, crinkling, and rugose [21,26,27,28]. Unlike many plant viruses, PVX lacks an invertebrate vector for transmission; instead, it spreads primarily through mechanical contact between healthy and infected plants, as well as contaminated tools, machinery, and personnel working in potato-cultivating areas [29,30]. Furthermore, the use of infected seed tubers for propagation accelerates the rapid dissemination of PVX disease throughout fields [29]. Potato virus X is a flexuous, filamentous, and non-enveloped virion particle, measuring approximately 470–580 nm in length and 12–13 nm in diameter [31,32]. Genetically, PVX is a single-stranded positive-sense RNA virus of approximately 6.4 kb (Figure 1). Its genome comprises a 5′ methylated cap, five ORFs, and a 3′ polyadenylated tail [29,33,34]. ORF 1 encodes a 166 kDa RNA-dependent RNA polymerase (RdRp), which contains domains of methyltransferase, helicase, and polymerase domains and is responsible for virus replication within the host cell cytoplasm [29].

The overlapping ORFs 2, 3, and 4 encode three proteins, TGB1 (25 kDa), TGB2 (12 kDa), and TGB3 (8 kDa), collectively known as the triple gene block module, which facilitates cell-to-cell movement of the virus in infected plants [35,36,37]. ORF5, located near the 3′ end, translates into 18–27 kDa coat protein (CP). While its primary function is encapsidation, the CP also plays a supportive role in movement and uncoating within host cells [29,30,38,39,40,41,42,43,44]. For species demarcation within Potexvirus, the International Committee on Taxonomy of Viruses (ICTV) has established gene-based sequence identity thresholds, ≥72% nucleotide or ≥80% amino acid identity in the CP or RdRp genes, rather than full-genome comparison (see details in the 9th ICTV report of 2011; Chapter: Genus Potexvirus; 32). However, ICTV does not currently provide any criteria for distinguishing strains of PVX.

Strains are generally defined as groups of viral isolates within the same species that possess distinct, stable, and heritable biological, serological, or molecular characteristics [45]. The classical systematic categorization of biological PVX strains was first proposed by Cockerham (1955, 1970), who assigned isolates into four groups (1–4) based on their ability to overcome three host resistance genes, Nx, Nb, and Rx, in potato cultivars [46,47,48,49]. Strain 1 isolates are susceptible to both hypersensitivity genes (Nx and Nb) and induce a hypersensitive response (HR) in inoculated plants. Strain 2 overcomes Nx but remains susceptible to Nb. Strain 3 overcomes Nb but triggers HR with Nx, while Strain 4 overcomes both Nx and Nb yet is restricted by the extreme resistance gene Rx. Distinctly, Köhler (1962) distinguished PVX subtypes based on their thermal inactivation points [50]. Subsequently, four major serological clusters of PVX isolates were identified using monoclonal antibodies targeting surface epitopes of CP [29,51]. Later, Komatsu et al. (2005) classified Japanese PVX isolates into two biological groups, “Common” and “Necrosis”, based on symptom expression and infection patterns in Mexican wild potato [52]. A significant shortcoming of the current PVX classification framework is the lack of standardized, full-genome-based demarcation criteria and a unified nomenclature system for both species and genomic strains. Such a framework is essential for comprehensively assessing genome-wide diversity and accurately inferring the genetic relationships among isolates. By contrast, the traditional pathotypic classification relies solely on biological characteristics, particularly the ability to overcome specific potato resistance genes, and therefore does not adequately reflect the evolutionary dynamics and phylogenetic relationships among PVX isolates.

Potato virus X exhibits a widespread global distribution. Previous phylogenetic analyses, based on 370 full-length PVX isolates, have established two primary phylogroups (I and II) and five subgroups (I-1, I-2, II-1, II-2, II-3), supported by 12 statistically significant clusters. While subgroups I-2 and II-3 are predominantly Andean, and II-1 is restricted to Europe, subgroups I-1 and II-2 comprise a mix of isolates from both regions [18]. The international trade of potato plants and seed tubers is recognized as a major contributing factor to its dissemination across the world [18,29]. Economically, PVX also imposes substantial losses on potato production, reducing yield by approximately 30–40% [39,40]. Under mixed infection conditions, particularly in synergistic combinations with potato virus Y (PVY), potato virus S (PVS), and potato virus A (PVA), the situation worsens considerably; affected plants display severe symptoms of rugose mosaic, crinkling of foliage, and stunting, with yield losses rising to nearly 80% [18]. In some cases, PVX remains latent, failing to induce any characteristic symptoms in infected host plants. To mitigate its economic impact, various control strategies have been adopted globally, including diagnostic methods using CP antibodies, quarantine measures, seed certification programs, and the identification of both natural and engineered tolerant potato varieties [53]. However, widespread distribution, genetic diversity, and adaptability to fluctuating environmental conditions present significant constraints to strict implementation of pathogen preventive strategies and developing effective antiviral resistance in potato. Despite being ranked among the ten most economically important plant viruses worldwide and serving as a prominent model system for studying viral replication, movement, and gene expression [54,55,56,57], research addressing the genetic diversity and evolution of PVX remains notably limited. There is an urgent need for comprehensive scientific investigations that would thoroughly analyze PVX recombination, phylogeny, and variability across complete genomes (population genetics) and in relation to host diversity and geographical distribution on a global scale [57,58]. This study focused on a comprehensive analysis of molecular identities, sequence demarcation criteria, population genetics, and evolutionary perspectives using 413 full-length PVX sequences available in the National Center for Biotechnology Information (NCBI) GenBank database [59]. The findings provided rigorous insights into pairwise sequence identities among PVX isolates, enabling the provisional revision of species and strain cut-off boundary values and the establishment of a putative strain-naming framework. The outcomes notably elucidated the extent of genetic diversity within the global PVX population, phylogenetic relationships between geographically sparse isolates, and recombination dynamics operating within the PVX genomes. Furthermore, the study enhanced our understanding of PVX geographical distribution and host range, enabling a thorough evaluation of its spread patterns, dominant genotypes, and adaptability. These insights are critical for assessing the virus impact on potato crops and developing sustainable resistance strategies against PVX.

## 2. Materials and Methods

### 2.1. Viral Sequences Retrieval and Alignment

The reference PVX sequence (D00344) was used as a query to retrieve genomic data through Nucleotide BLAST (BLASTn, National Library of Medicine (NLM), Bethesda, ML, USA) https://blast.ncbi.nlm.nih.gov/Blast.cgi?PROGRAM=blastn&PAGE_TYPE=BlastSearch&LINK_LOC=blasthome (accessed on 20 July 2025). A total of 413 full-length PVX genome sequences (approximately 6–7 kb) available in the NCBI GenBank database (National Library of Medicine (NLM), Bethesda, ML, USA) https://www.ncbi.nlm.nih.gov/nucleotide/ as of 31 July 2025 were retrieved in unaligned FASTA format [60,61]. These sequences were initially subjected to manual curation to remove redundant data, including duplicate sequences, partial genomes, coding sequences (CDSs), and cloning vectors. Subsequently, the associated biological metadata for all collected samples were documented. Similarly, 169 sequences from 51 other Potexvirus species were obtained. These sequences were aligned using the Multiple Sequence Comparison by Log-Expectation algorithm (MUSCLE, Robert C. Edgar, USA) version 5.3 implemented in Molecular Evolutionary Genetic Analysis (MEGA X, Sudhir Kumar, Institute for Genomics and Evolutionary Medicine, Philadelphia, PA, USA) version 12.0.11 (default parameter settings include gap opening penalty = −400, gap extension penalty = 0.00, cluster method = UPGMA, and maximum iterations = 16) [62]. Additionally, four partial PVX sequences from Pakistan (three CP and one TGB1) were downloaded. The metadata for all sequences, including accession numbers, nucleotide length, host, and geographic information, have been provided in Supplementary File S1: Tables S1, S2 and S4.

### 2.2. Similarity Calculation Among Full-Length PVX Genome Sequences

Pairwise nucleotide identities were calculated using Sequence Demarcation Tool (SDT; also known as Species Demarcation Tool, Muhire et al., Computational Biology group, University of Cape Town, Cape Town, South Africa) version 1.3, a freely available software used for calculating and visualizing pairwise sequence identity (https://web.cbio.uct.ac.za/~brejnev/) accessed on 1 September 2025, under the MUSCLE alignment algorithm, recognized as the most reliable method for percentage identity scoring along with Neighbor-Joining (NJ) tree-based clustering, for both the 413 full length PVX genomes and the 169 complete sequences from 51 other Potexvirus species (with default cut-off values set at 78% and 94%) [63,64]. A comparative genome identity analysis was conducted using SDT (with default cut-off values of 78% and 94%) for the four Pakistani PVX isolates against 34 full-length PVX genomes, together with the corresponding gene sequences representing diverse strains and geographical regions (metadata of 34 sequences available in Supplementary File S1: Table S1). The unaligned FASTA input file yielded a color-coded distance matrix and a distribution plot.

### 2.3. Gene-Wise Sequence Identity Estimation Among PVX Isolates

The five individual gene nucleotide sequences, RdRp, TGB1, TGB2, TGB3, and CP, were extracted manually from the 413 full-length PVX genomes using an unaligned FASTA file in MEGA X and subsequently translated into amino acids [62]. Separate SDT analysis was then performed for each gene at both the nucleotide and amino acid levels to calculate pairwise percentage identities using default boundary values (78% and 94%) [63,64]. Additionally, to further evaluate sequence identity within the CP gene, we exploited 219 complete CP sequences (nucleotide and protein) available on NCBI GenBank alongside the CP dataset extracted from the 413 full-length genomes and analyzed them with SDT (with default cut-off values of 78% and 94%). The GenBank metadata for these additional CP sequences, nucleotide and protein, have been provided in Supplementary File S1: Tables S8 and S9.

### 2.4. PVX Genetic Diversity Exploration

Genetic diversity and evolutionary dynamics of PVX were investigated by analyzing the 413 isolates with DNA Sequence Polymorphism (DnaSP, Julio Rozas Lab, University of Barcelona, Barcelona, Spain) version 6.12.03 [65]. The following parameters were estimated: nucleotide diversity (π), number of segregating sites (S), number of haplotypes (h) and haplotype (gene) diversity (Hd), and the average number of pairwise nucleotide differences among sequences (K) [66]. Additionally, population genetic differentiation was assessed using multiple indices, x^2^, Kst, Z, Fst, Gst, Nst, and Snn, along with gene flow (Nm) [67,68,69,70]. Before analysis, the aligned PVX sequences dataset was divided into two populations and afterward been analyzed under following parameter settings (analyzed region = 1–7726, sites with alignment gaps = excluded, premutation test replicates = 1000, and pseudorandom number seed = 2,966,463). To evaluate the robustness of population genetic estimates, three independent analytical analyses were performed using the same dataset of 413 PVX sequences under different grouping strategies. In the first analysis, sequences were divided according to geographical origin, with Population 1 (P1) comprising 348 isolates from South America and Population 2 (P2) including 65 isolates from Europe (25), Asia (20), Oceania (1), Africa (7), and North America (12). The associated metadata for each isolate is available in Supplementary File S1: Table S1. In the second analysis, sequences were randomly divided into two populations (P1 and P2) consisting of 207 and 206 isolates, respectively, as a control to evaluate the effect of arbitrary population assignment on genetic diversity, population differentiation, and gene flow. In the third analysis, the dataset was partitioned according to recombination status, with Population 1 (P1) comprising 363 non-recombinant genomes and Population 2 (P2) consisting of 50 recombinant genomes, to independently assess the contribution of recombination to global PVX diversity.

### 2.5. Phylogenetic Analysis of PVX Isolates

A phylogenetic analysis of the PVX isolates was conducted using the maximum likelihood method (ML, Joseph Felsentein, SEA, USA). The evaluation was performed with MEGA X version 12.0.11, using a general time reversible (GTR) nucleotide substitution model with 1000 bootstrap replicates (standard) based on the aligned sequences file [62,71,72]. Additional analysis parameters included nucleotide substitution type, uniform rate among sites, complete deletion of gaps, the ML heuristic method set to Nearest-Neighbor Interchange (NNI), the initial tree for maximum parsimony set to NJ/MP (default), and the number of computational threads set to 12. Mid-point rooting was applied for phylogenetic tree visualization without an outgroup. The resulting phylogenetic trees were visualized, annotated, and customized using the display and annotation tools available in the Interactive Tree of Life (iTOL, Ivica Letunic and Peer Bork, European Molecular Biology Laboratory, Heidelberg, Germany) version 7.6 [73].

### 2.6. Recombination Analysis of PVX

Recombination analysis was performed using Recombination Detection Program (RDP, Martin et al., University of Cape Town, Cape Town, South Africa) version 5 Beta 80 [74], which employs nine different phylogenetic, distance compatibility, and substitution statistical methods, namely RDP [75], GENECONV [76,77], BootScan [78,79], Maximum Chi Square (Maxchi) [80], Chimaera [81], Sister Scanning method (SisScan) [82], PhylPro [83], LARD [84], and 3Seq [85], under default settings (*p*-value ≤ 0.05, Bonferroni correction for multiple comparisons, linear sequence type, and number of permutations = 0). The aligned sequences file was uploaded as input, and the output was generated as an Excel spreadsheet comprising recombination breakpoints, recombinant sequences, minor and major parent sequences, and confirmation values from each of the nine methods. Recombination events supported by at least five methods were manually identified and tabulated. To validate these findings, Genetic Algorithm for Recombination Detection (GARD, Sergei L. Kosakovsky Pond, LJ, USA) version 0.2, a statistical method freely available to run via Datamonkey version 3 web server, was additionally employed with default parameters, including nucleotide data type, faster run mode, universal genetic code, site-to-site variation (rv) = none, rate of classes = 0 (not applicable), maximum breakpoints = 10,000, and minimum partition size = 823 bp to detect recombinant regions, exclusively using 413 full-length PVX genome sequences [86].

### 2.7. Analyses of PVX Geographical Distribution and Host Range

Microsoft Excel (Microsoft Office 2024, Microsoft Corporation, Redmond, WA, USA) [87] was used to analyze the GenBank metadata, specifically country, year, and host of isolation, for each complete PVX sequence, to determine the PVX’s global center of origin, geographical distribution, and host range (see details in Supplementary File S1: Table S1).

## 3. Results

### 3.1. Rational Sequence Demarcation Criteria for PVX

**Figure 2:**
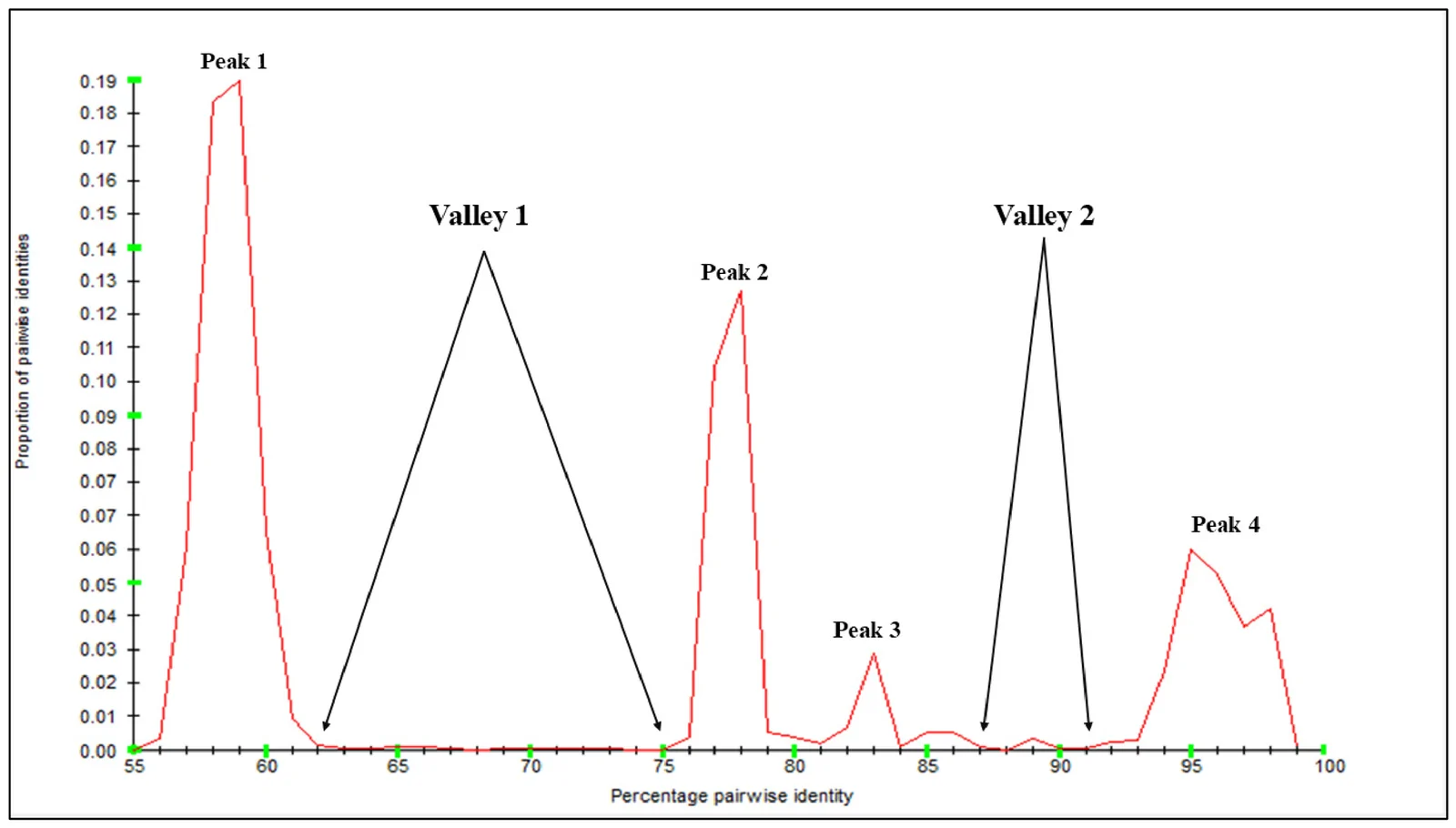
SDT analysis results of 582 complete genome sequences belonging to genus Potexvirus: Distribution plot indicating obvious peaks and valleys used for provisional species demarcation.

**Figure 3:**
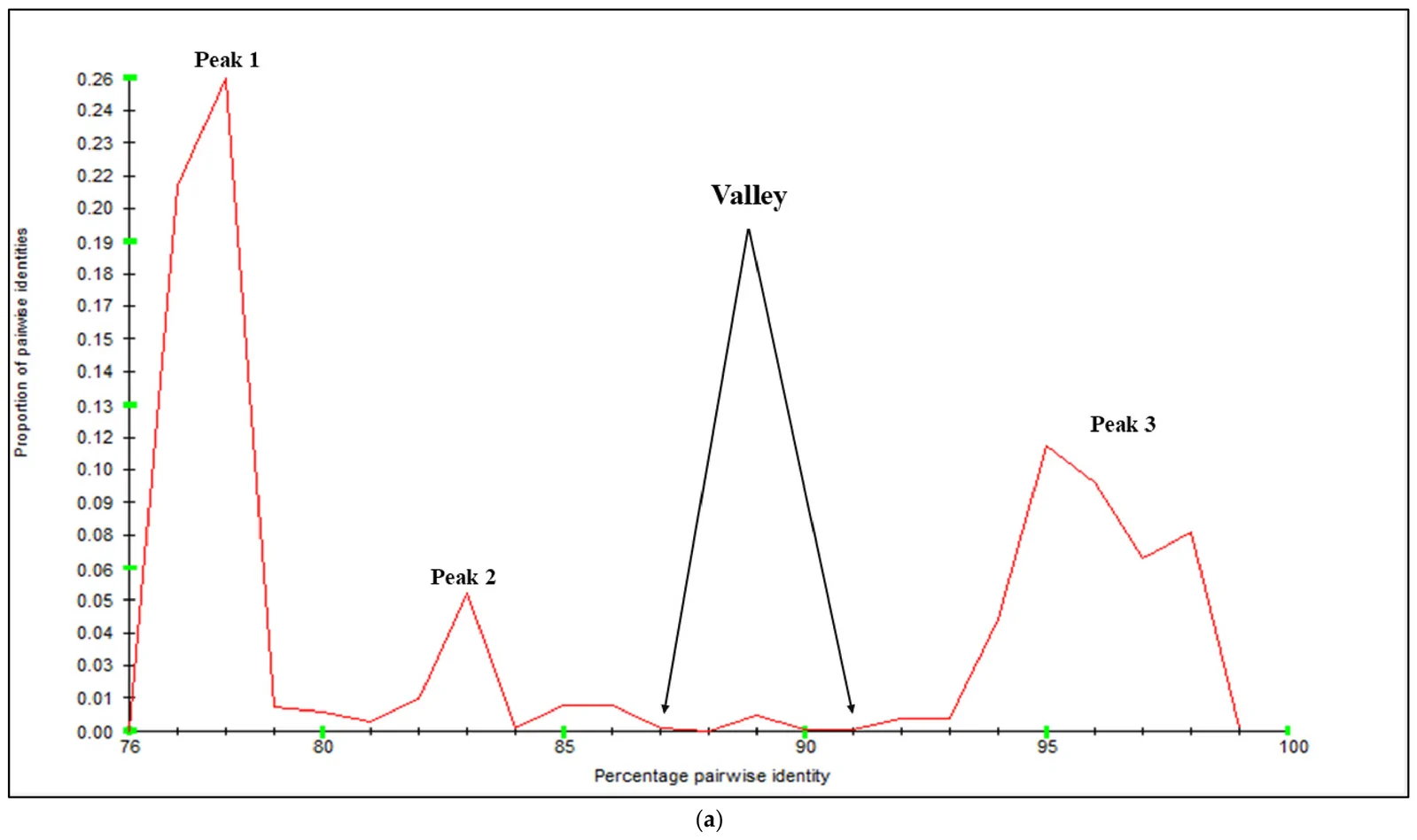

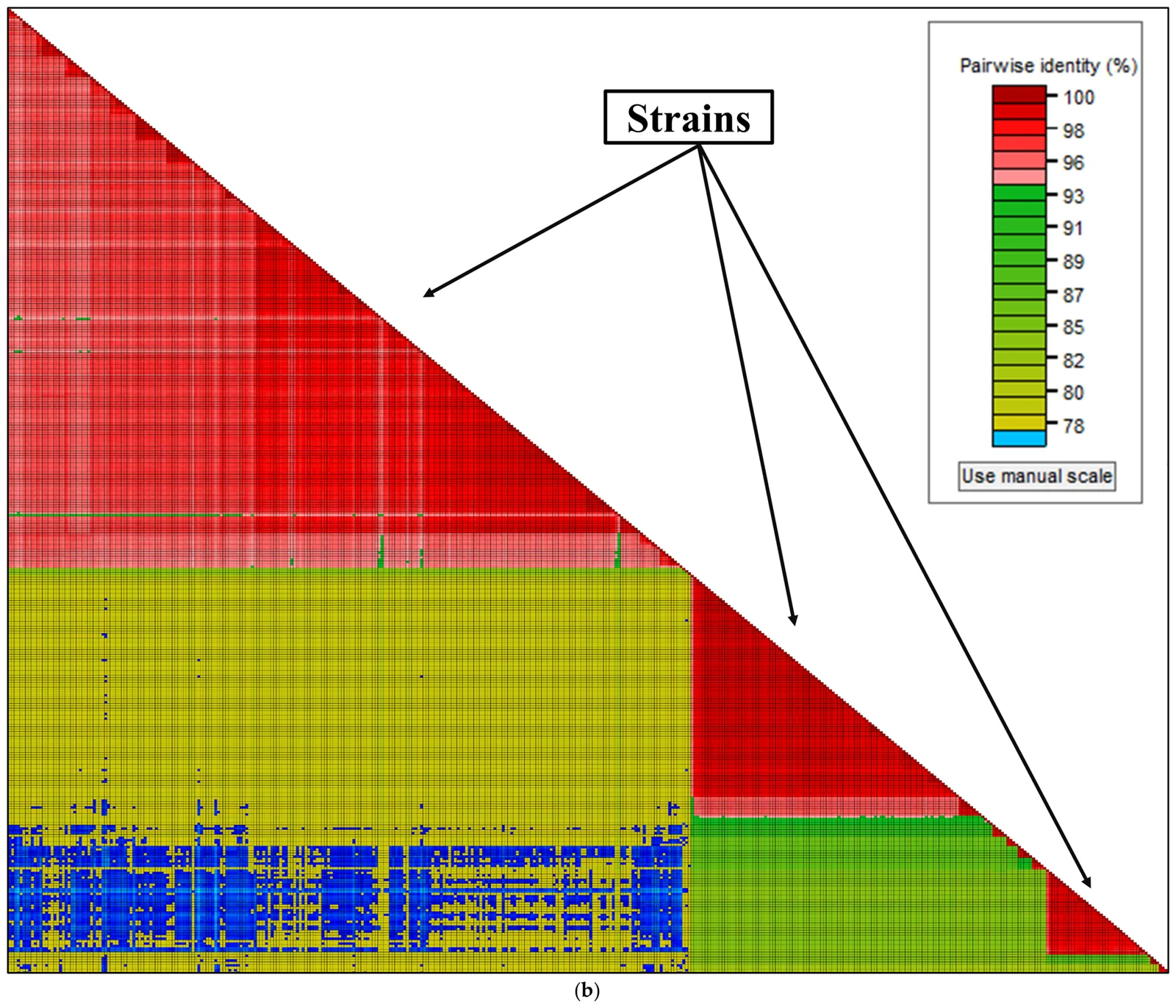
SDT analysis results of 413 PVX complete genome sequences: (**a**) Identities distribution plot representing obvious peaks and valleys used for provisional strain demarcation. (**b**) Color-coded matrix indicates genetically similar sequence groups based on three different colors (red, green, and blue); red indicates isolates sharing ≥ 94% pairwise identity, green mark isolates that share identity between 78–93% and blue represents sequences with <78% identity score.

**Figure 4:**
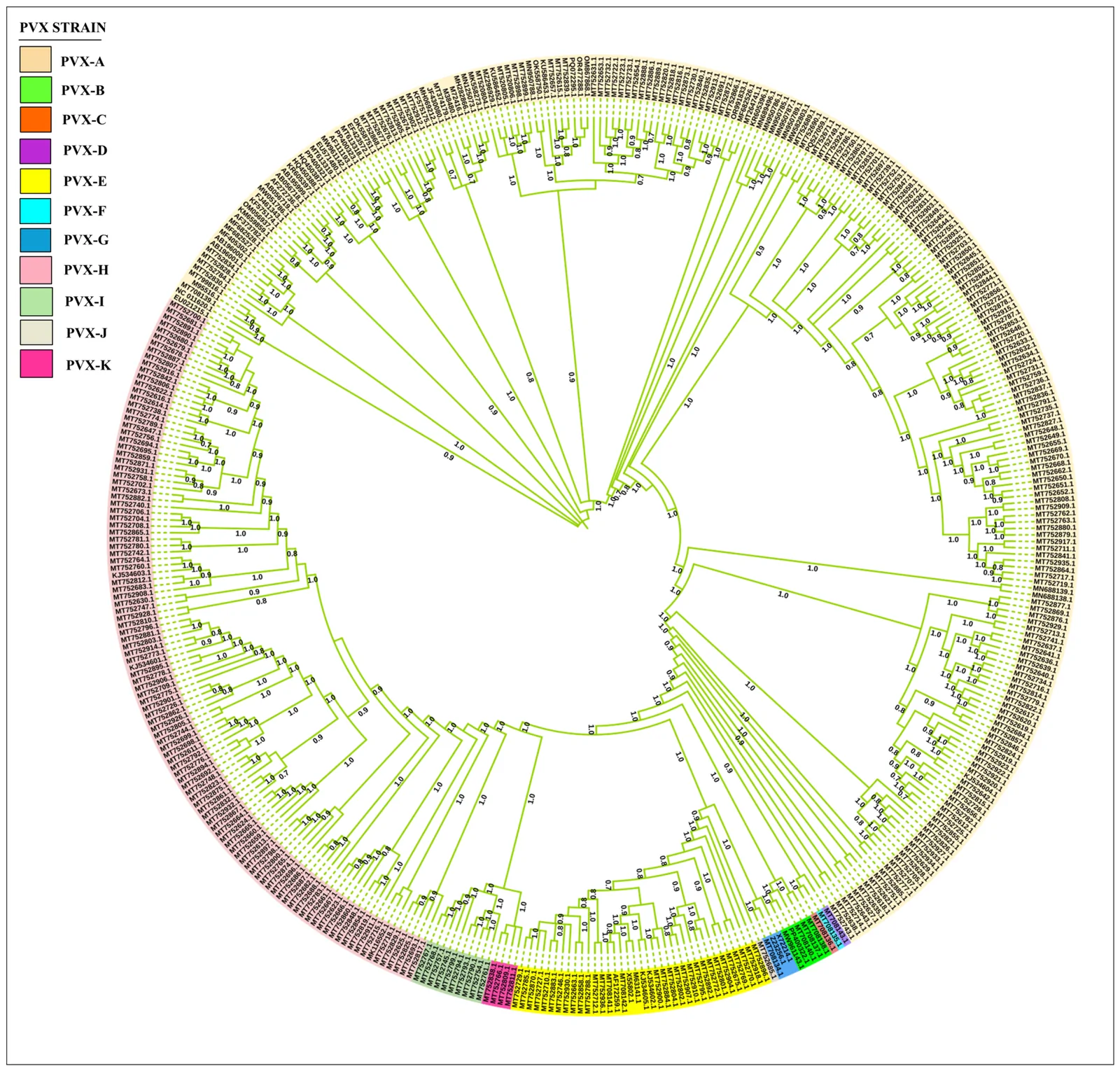
Maximum-likelihood (ML) method-based phylogenetic tree of potato virus X using 413 available full-length viral isolates. The evolutionary history was inferred using the general time reversible (GTR) nucleotide model implemented in MEGA X, and robustness of branches was supported with 1000 bootstrap replicates. Only bootstrap values above 70% are shown in the tree. All PVX isolates are identified by their GenBank IDs. The colors identifying the PVX phylogenetic groups are explained in the legend.

A total of 169,071 pairwise alignment scores were calculated using SDT from 582 full-length genomic sequences within the genus Potexvirus, comprising 413 PVX and 169 other Potexviruses, to establish an unambiguous tentative species demarcation threshold for PVX. The alignment data (Figure 2) delineated four distinct peaks, approximately 56–61%, 76–79%, 82–84%, and 93–99%, and two valley ranges, roughly 62–75% and 87–91%, in pairwise identities. These valleys, based on genome-wide identity, serve as demarcation thresholds that minimize classification conflicts, ensuring that isolates are assigned to proposed species or strain groups with the highest confidence. Additionally, pairwise alignments were performed using SDT on 413 full-length PVX genome sequences available in NCBI GenBank. This analysis generated a total of 85,078 pairwise identity scores, which served as the basis for reliable provisional strain demarcation of PVX [63,88,89,90,91]. The distribution of the identity scores across 413 sequences revealed three distinct peaks at approximately 76–79%, 82–84%, and 93–99% and one clear valley at approximately 87–91% identity (Figure 3). Based on the SDT pairwise identity distribution plot (Figure 3a) and color-coded matrix (Figure 3b) from 413 PVX genomic sequences, a tentative threshold of 89% nucleotide sequence identity was selected for provisional strain demarcation. This threshold grouped the 413 PVX sequences into eleven putative strains (Table 1), all showing ≥89% intra-group identity. Similarly, the analysis of the pairwise distribution plot (Figure 2) and nucleotide identities among isolates of 52 different Potexviruses supported provisionally adopt 76% species demarcation criteria for potato virus X (Percentage identities among 52 Potexvirus species are provided in Supplementary File S1: Table S3). All PVX isolates formed a tight cluster while showing clear divergence from all other sampled Potexviruses, thereby strengthening the whole-genome nucleotide identity of 76% as a preliminary sequence-based criterion for distinguishing PVX isolates from other Potexviral species. Accordingly, we propose that any unknown sequence sharing approximately 76% nucleotide sequence identity with one or more PVX isolates should be assigned to PVX [92]. Furthermore, all other Potexviruses also grouped distinctly at this species threshold, except for Asparagus virus 3 and Papaya mosaic virus, which exhibited high divergence. Strong statistical support from phylogenetic analysis (Figure 4) confirmed both the 76% species and 89% strain thresholds as a robust tentative criterion, with no apparent classification conflicts.

### 3.2. PVX Strain Naming

Although the ICTV has not established standardized guidelines for strain nomenclature [32], researchers have adopted diverse naming approaches in the literature. These include the following: (1) geographic origin (e.g., PVX-HB [93]); (2) symptoms and location (e.g., PVX-OS [52,94]); (3) country (e.g., PVX-Korea [95]); (4) city (e.g., PVX-Tula [41]); (5) research center or laboratory code (e.g., PVX-ROTH1 or PVX-GAF318-4.1 [96]); (6) genomic features (e.g., PVX-CP4 [97]); (7) pathotype classification (e.g., PVX-X3 [98]); and (8) host, location, and year (e.g., PVX-ptDel-9 [99]). In the current analysis, we thoroughly considered all previously employed naming approaches (see details in Supplementary File S1: Table S11 and Supplementary File S2: Figure S13). Several rational conflicts emerged: (a) Multiple previously classified PVX strains, including OS, BS, and ROTH1, clustered within a single putative PVX-A strain, indicating redundant nomenclature. Isolates from multiple genomic strains induced identical symptoms. For instance, both necrosis and mild mosaic were observed across all 11 genomic clusters. (b) Multiple putative strains originated from the same geographic location, with nine distinct PVX genomic strains reported from Peru alone. (c) Identical years of isolation were recorded across different putative strain groups. (d) Multiple provisional strains infected the same host species, with more than one PVX genomic lineage causing infections exclusively in potato.

To resolve these inconsistencies, we proposed a simple alphabetic nomenclature for PVX genomic strains following the precedent established for Mastrevirus (Table 1) [63]. The 11 putative PVX strain groups were ordered chronologically based on the earliest known isolate within each group; accordingly, the genomic strain containing the oldest isolate (from 1928) was designated PVX-A, the genomic strain with next oldest PVX isolate (from 1940) was named PVX-B, and so forth. In cases where the three strain groups shared identical isolation dates for their oldest isolates, we prioritized host differences first, followed by specific pathotype designation, to resolve the conflict.

For PVX isolates within a putative strain that share biological metadata (e.g., host, country, city, and year), we assigned an additional identifier based on the frequency of isolates from the same country, city, or area (Table 2; only five representative isolates per genomic strain are listed in Table 2, with the remainder provided in Supplementary File S1: Table S12). Further subdivision of PVX isolates below the strain level proved ambiguous, owing to the high nucleotide identity observed within each genomic cluster (see Supplementary File S1: Tables S13–S16).

### 3.3. Virus Nomenclature

It has been proposed that PVX isolates may be written in the following format: virus name followed by putative strain name separated by hyphen “-” and followed by isolate descriptor, as outlined below:

< Virus name ˃-< Strain name ˃ [< Country of isolation/Territory code ˃-< City/Location of isolation ˃-< Host of isolation/Lab Code/Old name ˃-< Year of isolation ˃].

The isolate descriptor, enclosed in square brackets (“[ ]”), should be placed immediately after the proposed species/strain name of the virus (Table 2). The first subfield of the descriptor should be a two-letter international country code indicating the country of isolation/origin, and the last subfield must always be the year of isolation [63,100]. Between these, additional subfields containing virus-related data may be included, such as city/district of isolation or host of isolation or lab code under which the sample was processed or the collector’s name or the previous isolate’s name or sample number. Each subfield must be separated by a hyphen “-” instead of “:”, which is typically used to indicate branch length information in the Newick phylogenetic tree file format (see en.wikipedia.org).

### 3.4. Gene-Wise Sequence Similarity Among PVX Isolates

All five gene sequences from the 413 full-length PVX isolates were individually analyzed using SDT software. Pairwise nucleotide and amino acid percentage identities were calculated to assess the variability within each gene and its corresponding protein product. The results (Table 3) revealed that all five PVX genes exhibited considerable identity at both nucleotide and protein levels (SDT plots for each analysis are provided in Supplementary File S2: Figures S1–S10). For the RdRp and CP genes, sequence identity remained within ICTV criteria (≥72% nucleotide or ≥80% amino acid identity with CP or RdRp) for PVX isolate classification. This was further validated by pairwise sequence identity analysis of 632 full-length CP genes, available in the databases (see details in Supplementary File S1: Table S10 and Supplementary File S2: Figures S11 and S12), which confirmed the ICTV-provided thresholds [18,53,101,102,103,104,105,106]. In contrast, the triple gene block ORFs displayed variable levels of conservation. Notably, the TGB3 gene showed comparatively lower sequence identities than TGB1 and TGB2.

## 4. Analysis of PVX Population Genetics

A total of 6042 bp from the complete genome of 413 PVX sequences were analyzed using DnaSP [107,108], after excluding alignment gaps (1684 nucleotide sites). This analysis was performed to estimate the genetic variation and evolutionary patterns present within the PVX population [109,110]. In the first analysis, the PVX dataset was grouped into Population 1, consisting South American isolates, and Population 2, comprising isolates from remaining regions, including Asia, Europe, North America, Oceania, and Africa, to minimize the impact of unequal sampling and enable a more balanced population genetic comparison. A control analysis was performed by randomly dividing the PVX isolates into two populations, whereas recombination was independently examined by comparing recombinant and non-recombinant genomes.

### 4.1. Genetic Diversity Analysis

Genetic diversity within the PVX population was evaluated using three independent analyses under different grouping strategies, including geographical populations, random populations, and recombinant versus non-recombinant populations. The detailed estimates from all three analyses are provided in Supplementary File S1: Tables S18, S21 and S24. Across all three analyses of the same PVX dataset (413 full-length isolates), the global PVX population consistently exhibited high genetic diversity, with 2851–2852 polymorphic sites (S), 408–410 haplotypes (h), haplotype diversity (Hd = 0.99994–0.99996), and nucleotide diversity (π = 0.12814–0.12815) (Table 4) [111,112,113,114,115]. Comparative analysis demonstrated that the general diversity estimates remained highly consistent across all grouping strategies. However, within the geographical analysis, Population 1 accumulated more mutations and exhibited higher genetic diversity with respect to π and Hd, suggesting that this group of PVX isolates harbors a broad spectrum of genetic diversity and a more complex evolutionary history than Population 2. In addition, the random and recombinant grouping provided overall comparable diversity values, indicating that the remarkably high genetic diversity is a stable and independent characteristic of the global PVX population rather than a consequence of the grouping strategy.

### 4.2. Genetic Differentiation and Gene Flow (Nm) Estimation

The genetic differentiation and gene flow (Nm) among the PVX populations were assessed using multiple indices under three different grouping strategies (Table 5 and Table 6), with detailed results provided in Supplementary File S1: Tables S19, S20, S22, S23, S25 and S26. Across all analyses, Chi-square, Hs, and Hst statistics supported the null hypothesis of no genetic differentiation (non-significant *p*-values), whereas Kst, Kst*, Z, Z*, and Snn statistics strongly rejected it (highly significant *p* values), indicating considerable nucleotide differentiation but limited haplotype differentiation (Table 5). Comparative analyses showed that geographical populations exhibited weak genetic differentiation (Gst = 0.00218) with a biologically meaningful gene flow rate (Nm = 114.26), whereas nucleotide and fixation indices revealed moderate differentiation (Nst = 0.19100 and Fst = 0.18375) and limited gene flow (Nm = 1.06–1.11), suggesting substantial genetic connectivity among geographically distinct PVX populations [111,112,113,114]. In contrast, the random population showed negligible genetic differentiation (Gst = 0.00004) with high gene flow (Nm = 7103.67), while Nst (0.59955) and Fst (0.58169) indicated strong genetic differentiation and very low rate of gene flow (Nm = 0.17–0.18), suggesting that the arbitrary distribution of population exaggerates population structure. Similarly, recombinant and non-recombinant populations exhibited weak genetic differentiation (Gst = 0.00326) and high estimated gene flow (Nm = 76.4), while Nst (0.37133) and Fst (0.36766) indices reflected moderate population structuring and gene flow (Nm = 0.42–0.43), suggesting that recombination contributes to population differentiation while maintaining genetic connectivity (Table 6). Collectively, these results depict a highly diverse and genetically connected global PVX population, although the extent of genetic differentiation and gene flow vary according to population grouping strategies.

### 4.3. Phylogenetic Analysis of Full-Length PVX Sequences

To evaluate their evolutionary relationships among PVX isolates, 413 complete genome sequences were investigated using MEGA X, and a maximum likelihood (ML) phylogenetic tree was constructed (Figure 4). The resulting midpoint-rooted tree displayed a plausible topology comprising five parallel basal lineages, with one lineage appearing to be the most ancestral. This ancestral clade most likely diverged into three subclades, from which the majority of potential PVX phylogenetic clusters originated. Within the tree, isolate groupings were strictly by strain (genotype), as all isolates from the distinct PVX genomic clusters formed tight, clearly delineated clusters with no overlap. These findings provide strong statistical support for a full-length genome-based classification system for PVX and validate the proposed demarcation threshold of 76% and 89% for PVX species and strain assignment, respectively [92]. Notably, the phylogenetic tree showed the clustering of earlier different strains within the same putative PVX strain group in current studies, with 13 of 17 previous strains grouped in genomic cluster PVX-A, while the strain lineages PVX-E and PVX-G contained two earlier strains each, indicating inconsistencies with existing literature (see Supplementary File S2: Figure S13).

Among the observed phylogenetic patterns, the four hypothetical basal lineages, along with two apparent sub-clades of the fifth lineage, evidently emerged into phylogenetic group PVX-A, which comprised most isolates (239) extracted from agricultural regions across Asia, Africa, Europe, Oceania, North America, and South America. The third sub-clade of the fifth lineage probably served as the primary ancestral source, giving rise to all inferred major PVX genomic clusters, including numerous sub-lineages of PVX-A. The phylogenetic lineage PVX- H was the second most populous (112 isolates), with its potential distribution confined to various regions of South America. Other genomic lineages, namely PVX-C (one isolate), PVX-D (one isolate), PVX-F (one isolate), PVX-I (10 isolates), PVX-J (one isolate), and PVX-K (four isolates), anticipated exclusive emergence in the Peruvian region. Meanwhile, the phylogroups PVX-E (36 isolates) and PVX-G (three isolates) likely originated in South America and Europe, whereas PVX-B (five isolates) tentatively evolved solely in Europe.

In contrast, the geographical distribution of PVX isolates showed no meaningful correlation with their phylogenetic placement. While the inferred data suggested that phylogroup PVX-A was globally distributed, other provisional genomic lineages, including PVX-C, PVX-D, and PVX-I, were seemingly confined only to the Peruvian region. Similarly, when examined in relation to host species, the phylogenetic distribution appeared equally incongruent, as the prime known host of PVX remains exclusively *Solanum tuberosum*. Collectively, these findings clearly indicate that PVX isolates should be classified based on their strain type rather than on geographic origin or host association [18,116].

### 4.4. Recombination Analysis of 413 Complete PVX Genomes

**Figure 5:**
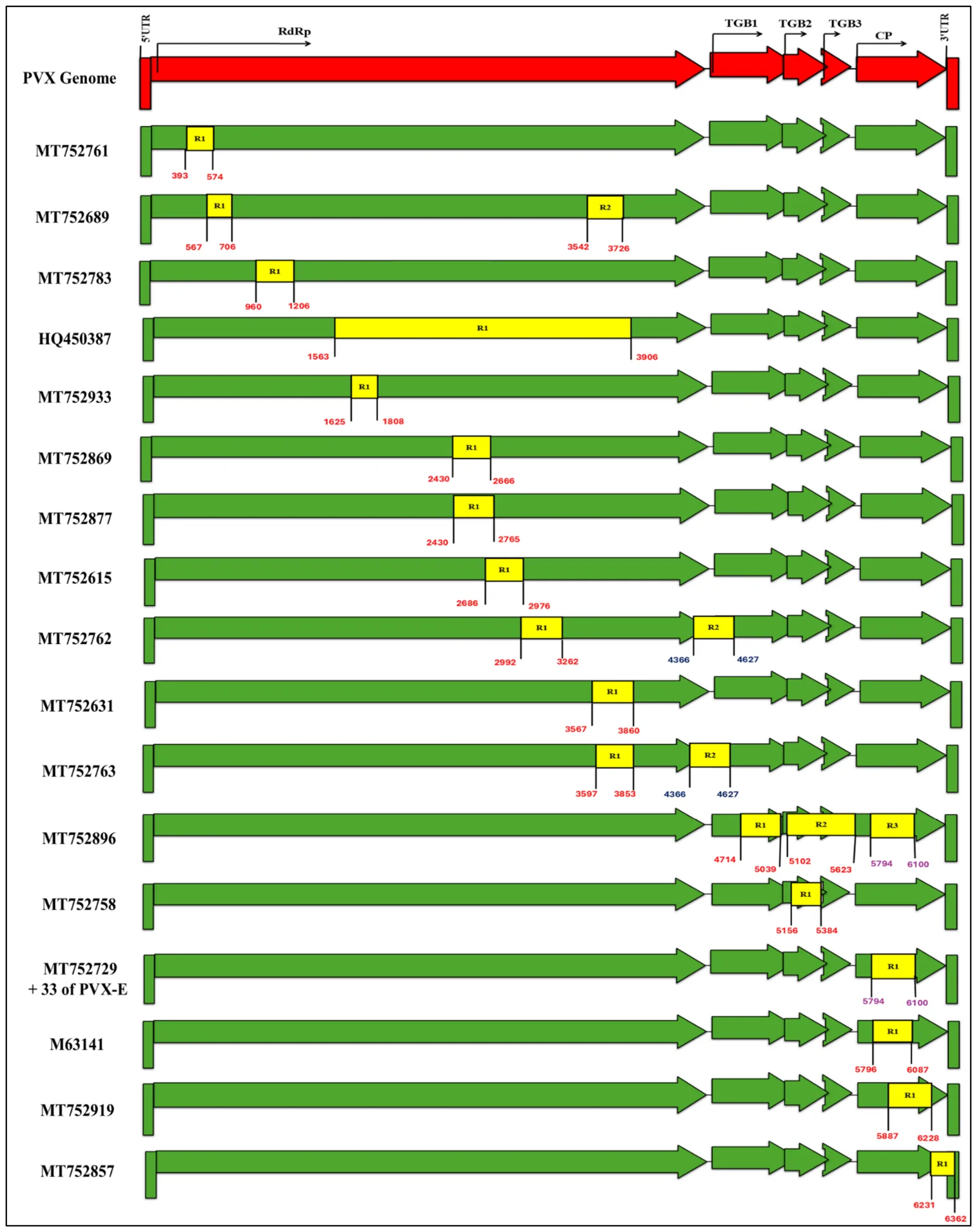
Recombination events reported in PVX using Recombination Detection Program (RDP). Details of each PVX recombinant, recombination events, parental sequences, and detection methods are provided in Supplementary File S1: Table S17. The green color in PVX genome indicates non-recombinant region and yellow color indicates the recombinant region (recombination breakpoint). Recombination breakpoints are denoted with different colors to represent unique and common recombination events among recombinant isolates; red indicates unique breakpoints, blue indicates the breakpoint (4366–4627) shared by two PVX-A isolates, and purple indicates the breakpoint (5794–6100) shared by 35 isolates of PVX-E.

Recombination analysis of 413 full-length PVX genome sequences was conducted using RDP, with only recombination events supported by five or more detection methods considered reliable. In total, RDP identified 20 distinct recombination events across 50 recombinant isolates, representing approximately one-eighth of the global PVX population (Figure 5). Although, most recombination events (18) were independently identified in sixteen different PVX genomes, the 35 isolates of PVX-E share a common breakpoint (5794–6100 bp), while the recombination event (4366–4627 bp) was identical among two isolates of PVX-A genomic strain. Of the 20 total recombination events, 12 were exclusively identified in the RdRp region, one in each of the TGB1 and TGB2 genes, and five independent breakpoints were detected in the CP coding region. Additionally, a single breakpoint was inferred at the junction of RdRp and TGB1, while another individual recombination event occurred in the intergenic region between TGB2 and TGB3 (Figure 5).

Geographically, 48 recombinant sequences originated from South America (46 from Peru, one from Argentina, and one from an unknown region of South America), while two recombinant isolates were collected from the UK and the USA, respectively (see detail of recombinants in Supplementary File S1: Table S17). These recombinant isolates were broadly distributed across multiple provisional PVX strains: PVX-A contained 11 recombinants, PVX-H contained two recombinants, PVX-I contained one, and notably, the entire PVX-E strain (comprising 36 isolates) was found to be recombinant. Two independent recombination events, R1 and R2, were detected in three PVX-A isolates (MT752689, MT752762, MT752763), whereas a single PVX-E isolate (MT752896) harbored three distinct recombinant breakpoints (R1, R2 and R3) within the genome (Figure 5). In comparison with previously published reports, 17 of the 18 recombinants documented in the literature were successfully detected in the current analysis [18,38,57]. Although many recombination events identified here supported the earlier findings, a few exceptions were observed, wherein breakpoints deviated from known patterns and appeared at novel genomic positions (see details in Supplementary File S2: Figure S14).

**Figure 6:**
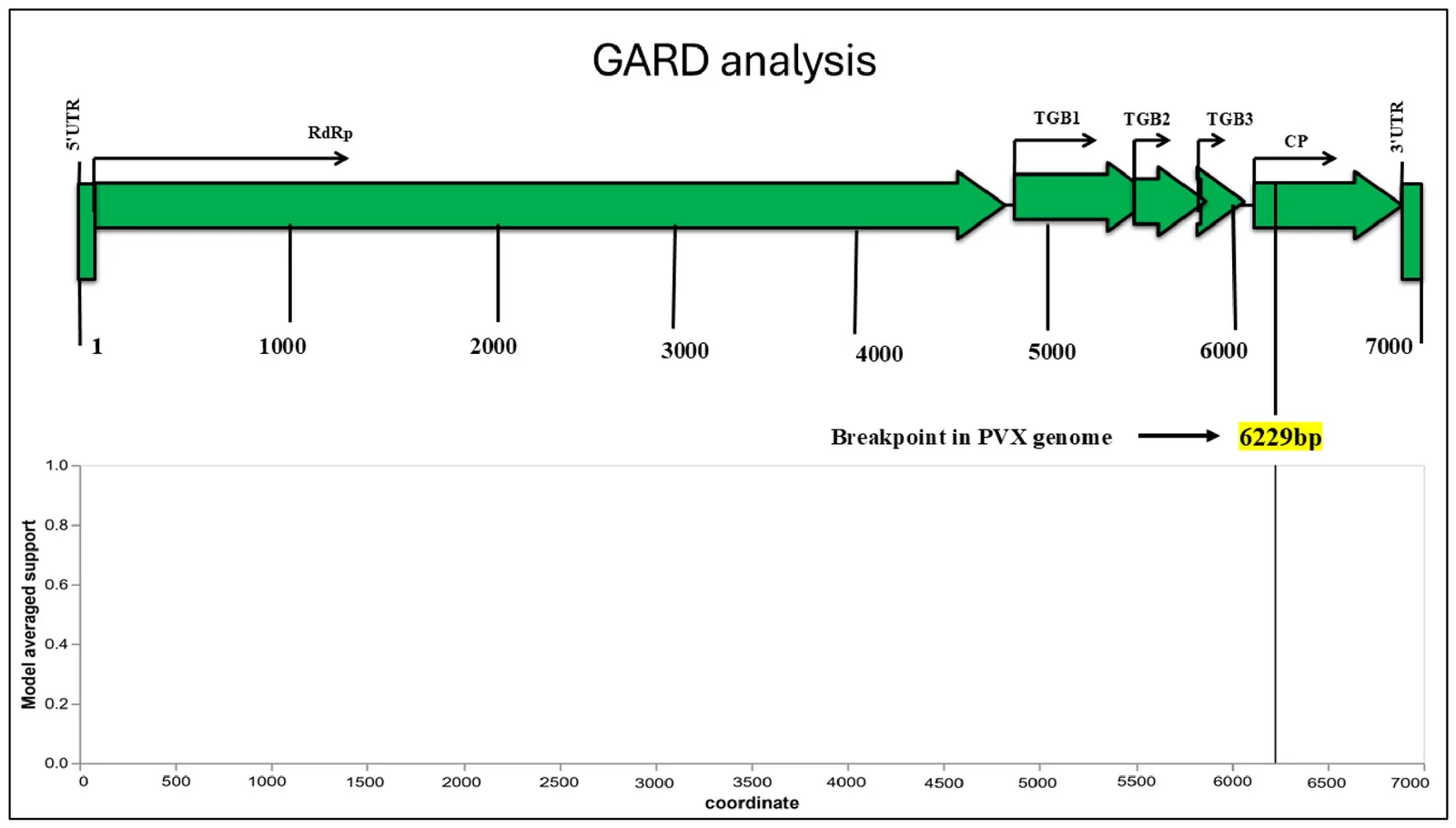
Recombinant region (hotspot) within PVX genome detected by Genetic Algorithm for Recombination Detection (GARD). The black solid line represents single recombination breakpoint detected at position of 6229bp within PVX genome.

Parental analysis revealed multiple parent-like sequences within the same PVX strain for most recombinant isolates, under both major and minor parent categories, a finding likely attributable to the high degree of genomic identity among these sequences (see Supplementary File S1: Table S17). One PVX-E isolate (M63141) from South America exhibited an unknown minor parent, a pattern consistent with earlier reports [18,38]. To minimize ambiguity, we preferentially adopted previously characterized parental sequences from the literature where applicable. For newly identified recombinants, the oldest sequence among all parent-like isolates was selected as the representative parental sequence [18,38,57]. Genome-wide analysis of 7726 aligned nucleotide positions (3627 sites of which are variable) across 413 PVX sequences was conducted using the GARD program. The disagreement in the aligned positions relative to the ideal PVX genome size appeared because two PVX isolates (MK558273 and M72416) comprised a genome sequence of approximately 7.5 kb. The GARD analysis identified only a single statistically significant breakpoint at position 6229 bp, located within the coat protein (CP) region of PVX (see best-fit model and total tree length plot figures in Supplementary File S2: Figures S15 and S16). However, the averaged likelihood plot (Figure 6) more broadly delineated a region between 5500 bp and 6500 bp as a recombination hotspot, validating only five of the 20 RDP-detected recombination events, corresponding to 38 recombinant PVX isolates [117].

### 4.5. Geographical Distribution and Host Range Analysis of PVX

Metadata associated with each full-length PVX sequence were analyzed to reconstruct the geographical distribution of PVX, thereby providing insights into its putative origin and global dissemination patterns. Additionally, the natural and diagnostic host range of PVX was characterized to elucidate its host specificity and experimental infectivity profiles.

### 4.6. Geographical Distribution of PVX Disease

**Figure 7:**
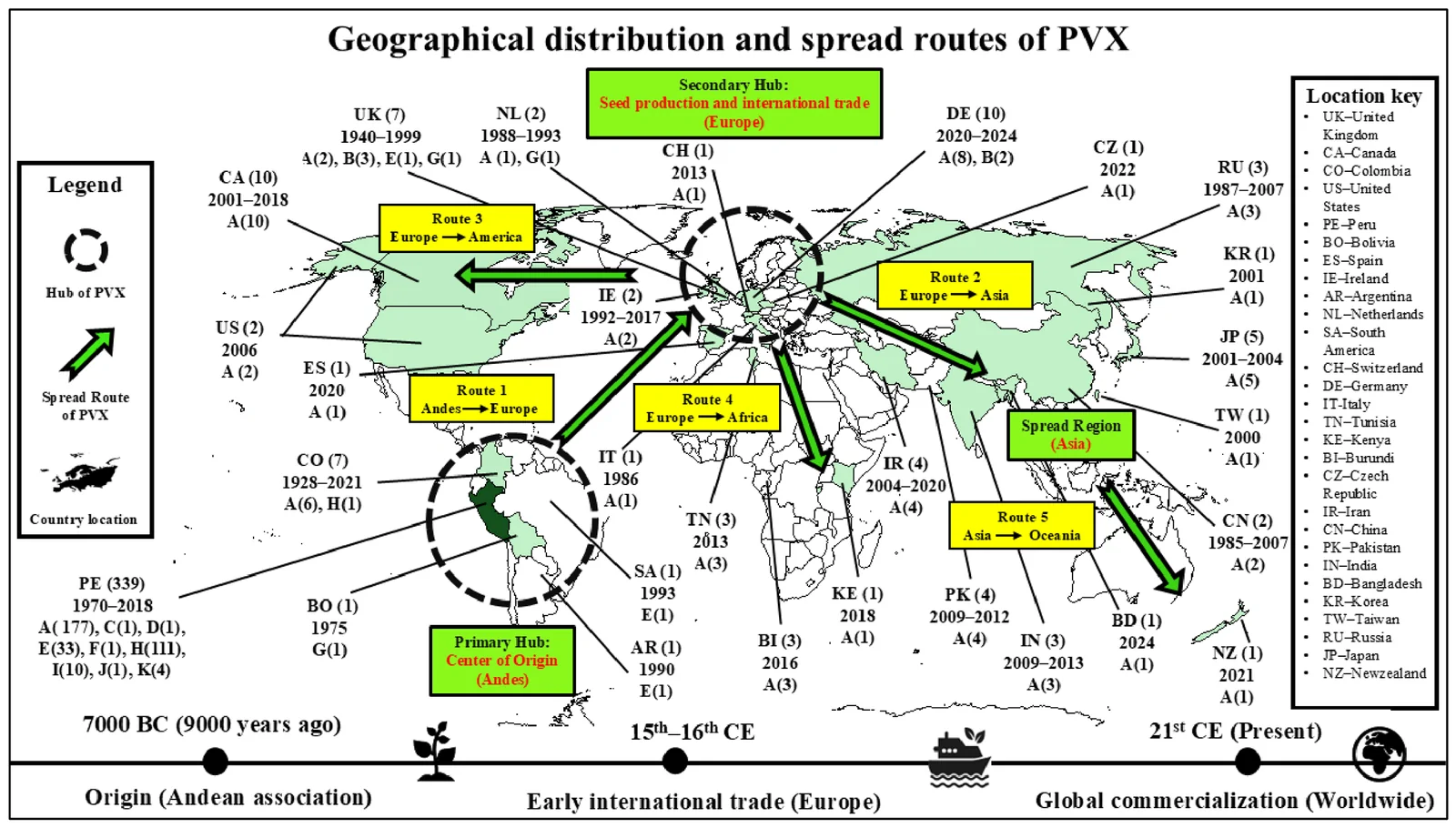
Geographical distribution of PVX across the globe. The PVX infected regions are selected with light to dark green color and each country is indicated by its two-letter international code followed by the total number of reported full-length PVX sequences, the isolation year of the first and lastly reported isolates and the isolate count of each PVX genomic strain reported from that country. The dotted circles and green boxes represent the regions with frequent genomic diversity of PVX. The green arrows and yellow boxes indicate the potential spread routes for global PVX dissemination. Partial PVX sequences reported from Pakistan indicated the regional distribution of PVX in South Asia.

The analysis showed that PVX exhibits a widespread geographical distribution, infecting major agricultural countries across the globe, including the United States, United Kingdom, China, Russia, India, Germany, and Canada. The distribution map (Figure 7) indicated clustering of PVX disease occurrences in regions characterized by intensive agricultural practices and further suggested potential routes of PVX dissemination [118,119,120,121,122,123]. The findings of phylogenetic diversity analyses were consistent with observed PVX geographical distribution and suggested the Andean region of South America as the probable origin of PVX. While the Andes has long been recognized for its historical association with potato cultivation, dating back to between 8000 BC to 5000 BC, recent molecular evidence from temporal analysis of PVX by Fuentes et al. (2021) supports the hypothesis that PVX emerged and diversified in South America approximately 9000 years ago [18]. During the mid-15th century CE, PVX infections are believed to have spread into Europe due to the import of seed potato from the Andes; however, the Irish famine of 1845–1849 may have subsequently led to the destruction of these potato cultivars in Europe [120,121,122]. The hypothesis further suggests that the reintroduction of Andean potato varieties in European regions via importation most likely once again facilitated PVX transmission into potato-growing countries of Europe. Subsequently, European countries potentially served as a secondary hub for PVX, where the virus may diversify extensively and could have spread to Asia and North America, followed by dissemination into Africa and Oceania through international trade of potato and germplasm [123,124]. Furthermore, our investigations suggested the predominance of genomic cluster PVX-A across global hotspots represented in the dataset. The Andean region exhibited the highest genomic diversity, with isolates belonging to all identified putative strain clusters except PVX-B. In contrast, the European region harbored infections of four PVX genomic strains including PVX-A, PVX-B, PVX-E, and PVX-G, whereas Africa, Asia, and Oceania were exclusively assigned to PVX-A, indicating comparatively limited genomic diversity in these regions. The SDT analysis of four partial PVX sequences from Pakistan [53,105,106] provisionally suggested their close genetic affinities with PVX-A isolates originating from Europe, Asia, and Oceania (see Supplementary File S1: Tables S5–S7).

### 4.7. Potato Virus X Host Range

**Figure 8:**
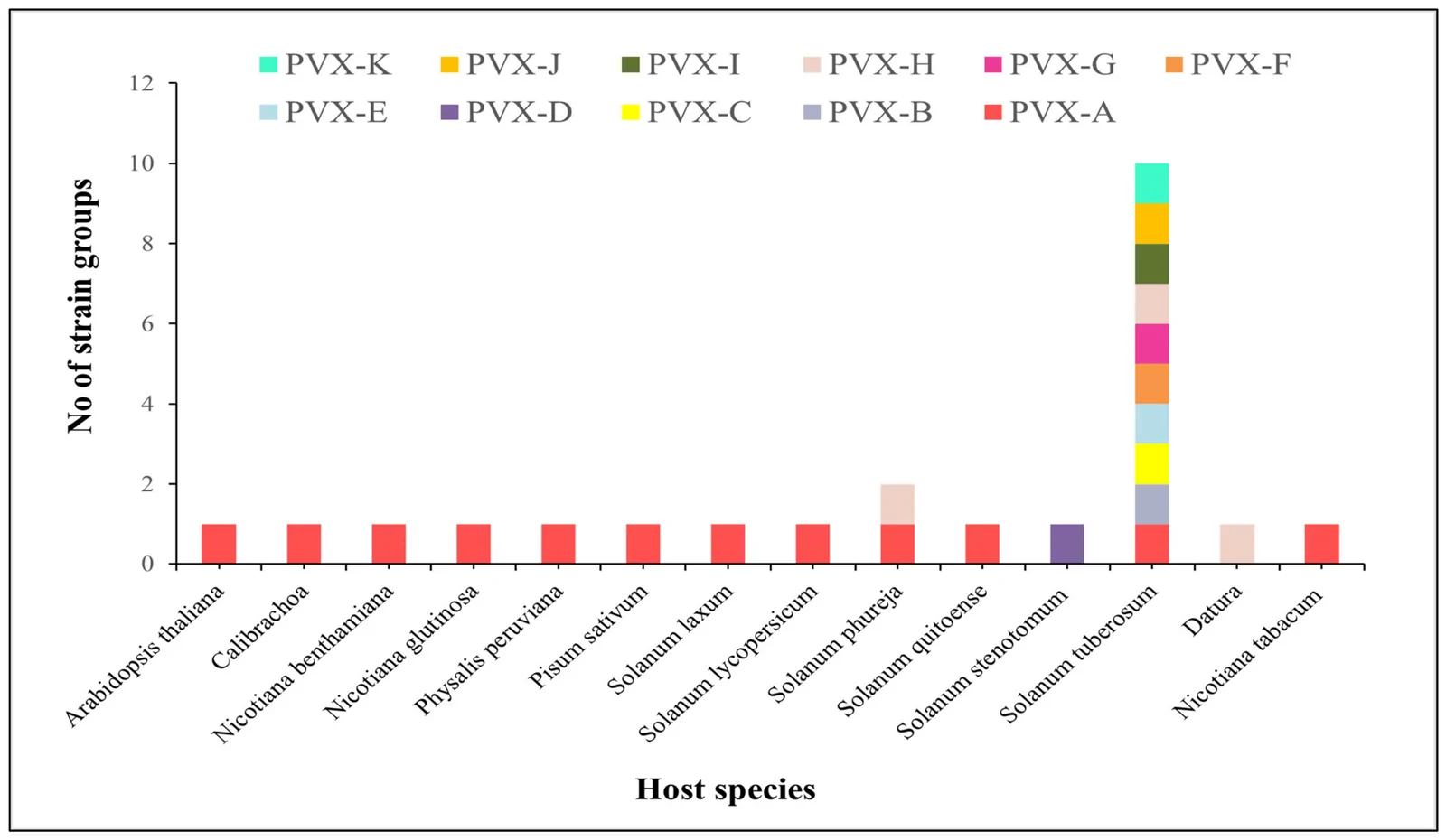
Host range of potato virus X.

Host range analysis revealed that PVX primarily infects 12 species within the *Solanaceae* family (see graph in Supplementary File S2: Figure S17). The data clearly identified *Solanum tuberosum* (potato) as the principal natural host species of PVX. Other naturally infected hosts within *Solanaceae* included *Calibrachoa*, *Datura*, *N. tabacum*, *N. benthamiana*, *N. glutinosa*, *P. peruviana*, *S. laxum*, *S. phureja*, *S. stenotomum*, *S. quitoense*, and *S. lycopersicum* [93]. In addition, PVX infection was also detected in *Arabidopsis thaliana* and *Pisum sativum*, which are key members of the *Brassicaceae* and *Fabaceae* families, respectively. The majority of PVX genotypes were isolated from potato, with PVX-A exhibiting the broadest host range among all strain groups (Figure 8). Experimental host plants inoculated under controlled conditions to characterize the biological features and infectivity spectrum of PVX isolates included *N. tabacum*, *N. benthamiana*, *N. occidentalis*, *Calibrachoa*, and potato micro-plants (see graph in Supplementary File S2: Figure S18). Collectively, the observed host diversity suggests that PVX possesses a moderate host range, although its primary linkage remains with potato [125].

## 5. Discussion

Potato ranks among the world’s most important food crops. Today, potato virus X (PVX) poses a significant agricultural challenge, causing substantial yield losses and threatening future global food security [30,126]. Although several studies have investigated the genomic diversity and evolutionary mechanisms of PVX, comprehensive insights into its genome-wide dynamics remain limited, constraining a deeper perspective of molecular evolution underlying its global prevalence [18]. In this study, we analyzed 413 full-length PVX isolates using a suite of bioinformatics approaches, encompassing sequence identities, population genetics, recombination patterns, phylogenetic relationships, host range, geographical distribution, inferred global spread routes, and proposed species and strain demarcation thresholds and nomenclature.

Although the ICTV has established gene-based species demarcation criteria for PVX, and several classical systems for strain classification are proposed, their applicability has become increasingly limited with the availability of a large number of full-length genomes and advances in comparative genomics. (See details in the 9th ICTV report of 2011; Chapter: Genus Potexvirus [32,46,47,48,49,50,51,52].) In the present study, we conducted sequence identity analysis on 413 full-length isolates of PVX using SDT with MUSCLE alignment to provisionally refine the species and strain demarcation criteria. We tentatively propose the cut-off value of ≥76% identity for PVX species and ≥89% identity for provisional strain classification, pending further validation using broader taxonomic, biological and epidemiological evidence (Figure 2 and Figure 3). A small number of outliers exhibited divergence from these thresholds, likely attributable to high nucleotide substitution rates in Potexviruses. To address such cases, we have proposed that a new isolate be assigned to the species or putative strain with which it shares the highest pairwise nucleotide sequence percentage identity [101]. Previous strain-naming approaches have been inconsistent due to overlapping based on year, area, and host of isolation; similar symptoms induced by isolates from multiple genomic clusters; and the clustering of most existing strains within one putative strain group (see Supplementary File S1: Table S11 and Supplementary File S2: Figure S13). The proposed alphabetical naming system eliminates ambiguity and provides a consistent framework (Table 2). This nomenclature is flexible and does not preclude future strain designations informed by consistent biological differences observed among members of distinct PVX genomic strains [63]. Further subdivision of the PVX population below the variant rank is not feasible due to the high degree of sequence homogeneity among the intra-strain members. This genetic identity may result from ongoing mutations, recombination, and gene flow within PVX and warrants further investigation to gain a deeper understanding (see Supplementary File S1: Tables S13–S16). The nucleotide identity thresholds established for members of *Geminiviridae* (begomoviruses) cannot be directly applied to members of family Alphaflexiviridae (PVX) because these virus families differ fundamentally in genome type, genomic organization, replication strategy, mutation rate, recombination dynamics, phylogenetic relationships, and host range. Consequently, family-specific and independently derived sequence identity criteria are required to achieve robust species and strain demarcation that accurately reflects their biological characteristics and evolutionary histories. In this context, full-genome-based analyses are particularly valuable, as they reflect the biological entities more accurately and provide a detailed view of the evolutionary forces driving virus evolution, including recombination, mutation, genetic drift, gene flow, and natural selection [117]. It is important to note that this study focused exclusively on provisionally establishing rational species and strain demarcation criteria for PVX using SDT; future work extending this framework to define species and strain boundaries for the entire genus Potexvirus would be a valuable continuation, though it lies beyond the scope of the present investigation. Moreover, future studies should explore biological, pathological, serological, and host resistance characteristics of proposed genomic lineages of PVX. Overall, the application of SDT in this analysis demonstrates its robustness and reliability, supporting its broader adoption in future sequence identity studies for other viral pathogens [88].

Investigation of gene-wise identity among PVX sequences revealed that the highest conservation within the PVX genome lies in the CP and RdRp regions, whereas greater variability exists within the TGB ORFs (Table 3). Although these findings align strongly with the ICTV’s gene-based classification criteria for PVX, they do not correspond well with sequence identity-based strain demarcation, rendering results ambiguous and invalidating strain classification based solely on gene-level data. An additional limitation is that this approach fails to capture the considerable genetic diversity present in the unexamined genomic regions, which may be essential for unbiased detection and for developing resistance against PVX disease. Gene-based detection analyses primarily support species-level distinction but are unable to specify the genotype of PVX infection in investigated host plants. Moreover, the phylogenetic tree constructed from gene sequences instead of complete viral genomes may introduce ambiguity regarding viral genome architecture and lead to incorrect evolutionary inferences [116]. Consequently, gene-based identity classification is inferior to full-length genome-based classification, serving only as a preliminary detection tool for early detection of infection, species differentiation, and gene-based molecular research. Future studies should prioritize identifying variable genomic regions across the 11 distinct PVX genotypes to improve understanding of genetic diversity and to advance preventive strategies for better disease management.

The present study reveals that the PVX exhibits extensive frequency genomic polymorphism, characterized by high nucleotide diversity (0.12814–0.12815) and haplotype diversity (0.99994–0.99996), rendering the analyzed genome highly diverse irrespective of grouping strategy (Table 4). This pattern suggests substantial genetic variation resulting from the accumulation of mutations during PVX evolution. The elevated variability observed in Population 1 of geographical populations indicates that the South American group may share closer relatedness with the basal lineage in PVX phylogeny, with long-term divergence, and that the remaining PVX evolutionary groups likely evolved from this ancestral pool, an interpretation completely consistent with previous diversity studies [57]. Furthermore, comparison of three grouping strategies demonstrates low-to-moderate genetic differentiation, coupled with detectable but method-dependent genetic connectivity (gene flow) among broadly defined PVX populations (Table 5 and Table 6). Moreover, the geographical population analysis revealed remarkable genetic linkage between worldwide PVX populations, whereas the random analysis highlighted the influence of arbitrary population assignment rather than natural evolutionary divergence. Additionally, the recombinant analysis suggested a passive role of recombination in shaping the global population structure of PVX. Overall, these findings suggest that the global distribution of PVX is likely driven by simple population expansion from South American clusters, possibly facilitated by human-mediated agricultural practices and the movement of infected plant material worldwide. Concurrently, geographical barriers, quarantine measures, and local adaptive responses may contribute to the moderate genetic structuring; substantial gene flow appears sufficient to counteract the effects of genetic drift, thereby reducing divergence and maintaining significant genetic connectivity among geographically distant PVX clusters. Collectively, this study provides evidence that gene flow and mutation rates are primary drivers, with occasional recombination preserving the genetic diversity, adaptive sustainability, long-term persistence, and evolutionary success of PVX.

Phylogenetic analysis enlightened important evolutionary insights and elucidated the genetic relationships among globally distributed PVX isolates. Strain-level differentiation achieved high-resolution discrimination of distinct PVX evolutionary groups proposed in this study (Figure 4), while clustering of earlier different strains within the same phylogenetic lineage further supports the proposed strain demarcation framework (see Supplementary File S2: Figure S13). The predominance of phylogroup PVX-A suggests that it likely constitutes a phylogenetically heterogeneous and provisionally successful genomic lineage, characterized by ongoing congruent evolution, and might serve as the primary source of diversification for recently emerged phylogenetic clusters. The early divergence of several hypothetical parallel lineages into the PVX-A genomic cluster further implies that the underlying genetic diversity within this lineage may confer selective advantages, such as enhanced adaptability, ecological fitness, or transmission efficiency, that have facilitated the sustained persistence of PVX over centuries. Our analysis also supports the hypothesis that PVX probably originated in the Andean region, the primary hub where potato cultivation began as a staple food during early human civilization. With the onset of evident transcontinental human movements and plant material transport in the 15th or 16th century CE, PVX was likely introduced to Europe, which subsequently served as a potential secondary hub. These two regions probably served as the major evolutionary niches for PVX diversification into extant genotypes, driven by the influence of prevailing evolutionary forces. The global distribution of PVX is merely explicable by the population expansion from European isolates, facilitated by the export of potato or seed tubers to international markets [18,118]. In 2025, Korkmaz et al. [127] showed that PVX isolates from Eastern Anatolia, Türkiye, did not form a geographically distinct cluster but instead displayed phylogenetic affinities with isolates from Hungary, India, and the USA. This observation is consistent with the geographically mixed clustering pattern identified in the present study and supports the conclusion that PVX phylogenetic relationships are not strictly structured according to country or region of origin. Overall, our findings support a model of coevolution of PVX with potato, largely supported by historical dispersal, mutation, and diversification mechanisms. Furthermore, provisional strain-based classification is recognized as the most robust approach for genotypic classification of PVX.

Recombination is a key evolutionary and persuasive process in viruses, contributing to the formation of new genomic combinations, unique haplotypes, and novel phenotypes that play a crucial role in the emergence of new strains with enhanced virulence, expansion of host range, evasion of host immunity, and breakdown of molecular resistance in host species [128,129,130,131]. RNA plant viruses are particularly prone to high recombination, as observed in potato virus Y, brome mosaic virus, tomato bushy stunt virus, and cucumber mosaic virus [132,133]. Moderate recombination has also been reported in members of the genus Potexviruses, including pepino mosaic virus [134]. Analysis of 413 full-length PVX sequences using RDP suggested that PVX exhibits moderate recombination, with a data-dependent estimated rate of approximately 12.1% (Figure 5). Although this rate is lower than observed in PVY or begomoviruses, potentially due to the absence of an insect vector or the stringent implementation of quarantine protocols in the international potato cultivating regions [135,136,137], recombination nevertheless presumably functions as a secondary evolutionary determinant alongside mutation and gene flow, contributing to the maintenance of high genetic diversity essential for PVX’s continued survival and adaptability. Furthermore, the detection of crossovers across the entire PVX-E strain group (Figure 5 and detail of recombinants in Supplementary File S1: Table S17) underscores the critical evolutionary role of ongoing recombination in PVX, which cannot be overlooked and warrants perpetual surveillance programs for comprehensive pathogen monitoring. The predominance of recombination events in South American isolates supports earlier findings that emphasize the significance of this region as the center of origin and proliferation for PVX [18,38,57]. The detection of a higher number of recombinants compared to earlier studies is likely attributable to the increased availability of genomic data, which indirectly reflects improvements in PVX detection and sequencing worldwide. The presence of multiple crossover sites in some isolates reflects complex evolutionary histories that require further investigation for a clear conceptual understanding. Meanwhile, observed differences in recombination breakpoints among the same PVX recombinants, or the absence of recombination relative to previous reports (see Supplementary File S2: Figure S14), can be primarily attributed to advances in the accuracy, efficiency, and sensitivity of RDP algorithms, which now detect previously unresolved recombination signals. Ambiguities in parental assignment for PVX recombinants (see Supplementary File S1: Table S17) probably arise from high intra-strain sequence similarities among isolates, which have previously constrained efforts to define variant demarcation boundaries. GARD analysis of the 413 full-length PVX genomes detected only a single breakpoint in the CP gene sequence (Figure 6), providing limited resolution of recombination patterns and failing to fully support the majority of recombination events reported by RDP. Overall, these findings highlight a significant role for recombination in PVX evolution and suggest that integrated analytical approaches are necessary to achieve a comprehensive understanding of recombination dynamics.

The findings of spatiotemporal analysis suggest that PVX probably originated in South America (Andean region), associated with the earliest stage of human civilization in the Andes. This hypothesis is supported by evidence of its dispersal into Europe at two different time points (Figure 7). Initially, PVX may have moved to Europe when the earliest potato trade between two continents commenced, facilitated by Spanish explorers in the late 15th or early 16th century. Subsequently, following the late blight epidemic of the 1840s, imports of seed potato cultivars from South America may have reintroduced PVX to European countries, with the contemporary European PVX population likely representing descendants of these historical isolates, a scenario entirely consistent with earlier findings. Extensive potato cultivation practices and human-mediated international export of germplasm, seed tubers, and potato from Europe may have further entrenched the virus in Asia, North America, Africa, and Oceania [120,121,122]. Upon critically reviewing the global prevalence of genomic lineage PVX-A, it is worth hypothesizing that this genotype may be the oldest, having diverged earliest in the evolutionary history and sharing close relationships with common ancestors. It is also speculated that this putative strain is likely to exhibit significant evolutionary activity, including high mutation rates, substantial genetic connectivity, selection pressure, and homologous recombination, which underscores the successful global establishment of PVX-A and positions it as a potential ancestral stock for all other recently evolved phylogroups. The close identity between PVX isolates of Pakistan and PVX-A isolates reported from various countries of the world (see Supplementary File S1: Tables S5–S7) supports the hypothesis that PVX might have spread from Europe to Asia and subsequently extended to Oceania. Our provisional gene-based data further provide comprehensive insights into the regional distribution of PVX-A in South Asia, including Pakistan [21], where the virus appears to share common ancestry with isolates from neighboring countries. Overall, this study reinforces that human transport and the international movement of plant materials, particularly potato germplasm and tubers, are possible drivers for intercontinental and regional PVX, consistent with previous studies. Although a rising trend in PVX sequencing has been observed since the 21st century, more extensive sampling of PVX strains from underrepresented geographical regions is still required for an in-depth understanding of existing uncertainties. Future studies will also be essential to effectively elucidate the complexities of regional PVX diversity and to refine global distribution patterns.

Host range analysis confirms that PVX exhibits a moderate host range, with *Solanum tuberosum* (potato) serving as its primary host. PVX demonstrates significant adaptability, infecting multiple alternate hosts across the three different plant families, including *Solanaceae*, *Fabaceae*, and *Brassicaceae*. PVX also widely infects various international and local potato cultivars, showing no evident preference for specific potato genotypes. Among the strains, PVX-A displays the broadest adaptability, while the majority of PVX genotypes remain largely restricted to potato (Figure 8). Additionally, the study identifies *Nicotiana tabacum* as the most reliable diagnostic host of PVX, predominantly used in the laboratory for virus detection assays. Evaluation of experimental data related to natural and diagnostic host ranges provides comprehensive insights into the biological and epidemiological behavior of PVX under both field and glasshouse conditions [138]. However, future studies should focus on exploring strain-specific pathogenicity across different host plants to elucidate the host-virus interactions at the molecular level, enabling relevant authorities to develop more targeted and comprehensive disease management strategies and genetic engineering-based resistance development in suitable potato cultivars that are crucial for minimizing the potential impacts of PVX on potato cultivation and precision agriculture.

## 6. Conclusions

In this study, we propose provisional full-length genome-based classification criteria for PVX species (76%) and strain demarcation (89%), representing a novel and comprehensive approach not previously documented for members of the genus Potexvirus. Based on a tentative, dataset-dependent genome-wide identity threshold, the PVX isolates were systemically classified into 11 distinct genomic strains, which were subsequently organized and named alphabetically. Phylogenetic analysis strongly supports the distribution of PVX isolates into provisional genomic lineages over classification by geographical distribution or host range. Our findings further reveal that PVX is a highly diverse and evolutionarily dynamic potato virus, exhibiting only moderate genetic differentiation among geographically distinct populations, and identify diversity patterns consistent with the Andes and Europe as major hotspots contributing to the global emergence and diversification of PVX. The rigorous outcomes of this research will provide worthwhile insights into PVX population structure, evolution, and spread patterns to plant virologists and molecular biologists, facilitating the future development of molecular resistance against potato virus X to ensure sustainable potato production and to minimize its devastating impacts on food security, the agricultural sector, and global commerce.

## Figures and Tables

**Table 1:** Details of pairwise nucleotide percentage identities among 11 putative PVX strains. The bold diagonal values indicate the intra-strain identities among isolates of PVX.

PVX Strains (Number of Isolate)	Pairwise Nucleotide Percentage Identities										
	PVX-A (239)	PVX-B (5)	PVX-C (1)	PVX-D (1)	PVX-E (36)	PVX-F (1)	PVX-G (3)	PVX-H (112)	PVX-I (10)	PVX-J (1)	PVX-K (4)
PVX-A (239)	**92.1–100%**										
PVX-B (5)	77.5–79.1%	**98.0–100%**									
PVX-C (1)	83.0–84.6%	78.3–78.5%	**100%**								
PVX-D (1)	82.4–84.0%	78.1–78.3%	83.30%	**100%**							
PVX-E (36)	76.8–79.6%	80.8–81.9%	77.9– 78.6%	78.0– 79.0%	**95.2–100%**						
PVX-F (1)	82.7–83.8%	77.9–78.1%	87.60%	83.60%	77.8–78.6%	**100%**					
PVX-G (3)	77.8–79.5%	80.9–81.2%	77.9– 78.1%	78.2– 78.4%	86.8–87.8%	78.4– 78.6%	**99.2–100%**				
PVX-H (112)	77.3–81.1%	80.0–82.1%	77.6– 78.9%	78.2– 79.1%	82.7–86.4%	77.8– 78.7%	83.6–84.3%	**89.4–100%**			
PVX-I (10)	77.3–83.1%	81.0–81.5%	78.2– 79.1%	77.9– 78.4%	83.1–84.4%	78.1– 78.8%	82.9–83.4%	85.8–87.4%	**91.9–100%**		
PVX-J (1)	77.8–79.0%	81.4–81.7%	78.20%	78.30%	86.9–87.7%	77.80%	84.1–84.3%	82.3–82.7%	82.3–82.7%	**100%**	
PVX-K (4)	77.5–80.4%	80.6–80.9%	77.9– 78.0%	78.4– 78.5%	83.5–84.3%	77.9– 78.1%	83.1–83.5%	85.5–86.5%	87.2–88.3%	82.4–82.5%	**98.8–100%**

**Table 2:** Details of viral isolates that have been assigned to proposed strain clusters within species potato virus X.

Sr No.	Isolate Accession Number	Isolate Name	Acronym
1	NC_011620	Potato virus X-A [Netherlands-Nicotiana tabacum]	PVX-A [NL-N. tabacum]
2	MT708139	Potato virus X-A [United Kingdom-1980]	PVX-A [UK -80]
3	MT799816	Potato virus X-A [Spain-Nicotiana benthamiana-2020]	PVX-A [ESP-N.benthamiana-20]
4	M95516	Potato virus X-A [Ireland]	PVX-A [IR]
5	OK558751	Potato virus X-A [Canada-1-2001]	PVX-A [CA-1-01]
6	MW961143	Potato virus X-B [01]	PVX-B [01]
7	PP400322	Potato virus X-B [02]	PVX-B [02]
8	MT708140	Potato virus X-B [United Kingdom-Scotland-1940]	PVX-B [UK-SCT-40]
9	MT708137	Potato virus X-B [United Kingdom-England1-1983]	PVX-B [UK-ENG1-83]
10	MT708138	Potato virus X-B [United Kingdom-England2-1983]	PVX-B [UK-ENG2-83]
11	MT708136	Potato virus X-C [Peru-1970]	PVX-C [PE-70]
12	MT708143	Potato virus X-D [Peru-1973]	PVX-D [PE-73]
13	MT752896	Potato virus X-E [Peru-Lima1-2017]	PVX-E [PE-LIM1-17]
14	MT752663	Potato virus X-E [Peru-Cajamarca1-2016]	PVX-E [PE-CJA1-16]
15	MT752918	Potato virus X-E [Peru-Puno1-2017]	PVX-E [PE-PUN1-17]
16	MT752802	Potato virus X-E [Peru-Junin1-2016]	PVX-E [PE-JUN1-16]
17	MT752900	Potato virus X-E [Peru-Lima2-2017]	PVX-E [PE-LIM2-17]
18	MT708135	Potato virus X-F [Peru-1973]	PVX-F [PE-73]
19	MT708134	Potato virus X-G [Bolivia-1975]	PVX-G [BO-75]
20	Z23256	Potato virus X-G [United Kingdom-Harpenden]	PVX-G [UK-AL5]
21	X72214	Potato virus X-G [Netherlands-1993]	PVX-G [NL-93]
22	MT752783	Potato virus X-H [Peru-Ica1-2017]	PVX-H [PE-ICA1-17]
23	MT752865	Potato virus X-H [Peru-Junin1-2016]	PVX-H [PE-JUN1-16]
24	MT752706	Potato virus X-H [Peru-Huancavelica1-2018]	PVX-H [PE-HUV1-18]
25	MT752704	Potato virus X-H [Peru-Huancavelica2-2018]	PVX-H [PE-HUV2-18]
26	MT752742	Potato virus X-H [Peru-Huanuco1-2016]	PVX-H [PE-HU1-16]
27	MT752799	Potato virus X-I [Peru-Junin1-2016]	PVX-I [PE-JUN1-16]
28	MT752745	Potato virus X-I [Peru-Huanuco1-2016]	PVX-I [PE-HU1-16]
29	MT752767	Potato virus X-I [Peru-Ica1-2017]	PVX-I [PE-ICA1-17]
30	MT752768	Potato virus X-I [Peru-Ica2-2017]	PVX-I [PE-ICA2-17]
31	MT752757	Potato virus X-I [Peru-Ica3-2017]	PVX-I [PE-ICA3-17]
32	MT752685	Potato virus X-J [Peru-Cusco-2016]	PVX-J [PE-CUZ-16]
33	MT752809	Potato virus X-K [Peru-Junin1-2016]	PVX-K [PE-JUN1-16]
34	MT752838	Potato virus X-K [Peru-Junin2-2016]	PVX-K [PE-JUN2-16]
35	MT752766	Potato virus X-K [Peru-Ica-2017]	PVX-K [PE-ICA-17]
36	MT752811	Potato virus X-K [Peru-Junin3-2016]	PVX-K [PE-JUN3-16]

**Table 3:** Details of pairwise nucleotide and amino acid percentage identity scores of all five gene sequences among 413 PVX isolates.

Gene Sequence	Nucleotide Identities	Amino Acid Identities
RdRp	77–100%	89–100%
TGB1	74–100%	86–100%
TGB2	79–100%	84–100%
TGB3	52–100%	65–100%
CP	77–100%	86–100%

**Table 4:** Genetic diversity within PVX populations.

Population	No of Sequences	S	h	Hd	K	Pi(π)
Geographical Populations	413	2851	408	0.99994	774.23313	0.12814
Random Populations	413	2852	410	0.99996	774.26422	0.12815
Recombinant vs. non-recombinant	413	2852	410	0.99996	774.26522	0.12815

S = number of polymorphic sites, h = number of haplotypes, Hd = haplotype diversity, K = average number of pairwise nucleotide differences among sequences, Pi(π) = nucleotide diversity.

**Table 5:** Genetic differentiation among PVX populations.

Differentiation Index	Geographical Population	Random Population	Recombinant vs. Non-Recombinant
	Values		
x^2^	405.459, (df = 409), ns	413.000, (df = 409), ns	413.000, (df = 409), ns
Hs	0.99990, ns	0.99993, ns	0.99993, ns
Hst	0.00004, ns	0.00004, ns	0.00001, ns
Ks	736.62230	455.97607	693.92829
Kst	0.04858, ***	0.41109, ***	0.10276, ***
Kxy	769.86350	1091.71400	1024.25100
Ks*	6.15117	5.60220	6.03572
Kst*	0.01303, ***	0.10116, ***	0.03160, ***
Z	40,500.10777, ***	27,097.42643, ***	38,226.12395, ***
Z*	10.26863, ***	9.80505, ***	10.17123, ***
Snn	0.95400, ***	0.92736, ***	0.96126, ***
Dxy	0.12742	0.18069	0.16952
Da	0.02341	0.10510	0.06233

Probability obtained by the permutation test with 1000 replicates (ns = not significant; * = 0.01 < *p* < 0.05; ** = 0.001 < *p* < 0.01; *** = *p* < 0.001), x^2^ = Chi Square test, Hs = haplotype diversity within population, Hst = genetic differentiation among populations (haplotype-based), Ks = average nucleotide diversity within population, Kst = nucleotide differentiation among populations (sequence-based), Ks* = standardized nucleotide diversity within population, Kst* = standardized nucleotide differentiation among populations, Z = average divergence between populations, Z* = standardized divergence, Snn = nearest-neighbor statistic, df = degree of freedom, Dxy = average nucleotide divergence between populations, Da = net divergence excluding within-pop variation, Kxy = average number of nucleotide differences between sequences from two populations.

**Table 6:** Gene flow (Nm) among PVX populations.

Differentiation Index	Geographical Populations		Random Populations		Recombinants vs. Non-Recombinants	
	Value	Nm	Value	Nm	Value	Nm
Gst	0.00218	114.26	0.00004	7103.67	0.00326	76.4
DeltaSt	0.00642	4.73	0.05274	0.36	0.01351	2.12
GammaSt	0.05021		0.41252		0.10564	
Nst	0.19100	1.06	0.59955	0.17	0.37133	0.42
Fst	0.18375	1.11	0.58169	0.18	0.36766	0.43

Gst = coefficient of gene differentiation, DeltaSt = difference in nucleotide diversity within and among Populations, GammaSt = proportion of nucleotide diversity among populations, Nst = sequence differentiation index, Fst = fixation index, Nm = number of migrants per generation (gene flow).

## Data Availability

The original data presented in this study is contained within the article or Supplementary Material. Further inquiries can be directed to the corresponding author.

## Supplementary Materials

The following supporting information can be downloaded at https://www.mdpi.com/article/10.3390/v18090968/s1: File S1: Spreadsheet version of tables, including Table S1: Biological metadata of 413 full-length potato virus X isolates, Table S2: Biological metadata of 169 full length isolates of 51 Potexviruses, Table S3: Details of pairwise nucleotide identities among 52 different virus species of Genus Potexvirus, Table S4: Biological metadata of four partial PVX sequences from Pakistan, Table S5: Details of nucleotide percentage identities of four partial PVX sequences from Pakistan with 34 full-length isolates of proposed 11 PVX strains found in different geographical locations, Table S6: Details of nucleotide identities among CP sequence of three PVX isolates from Pakistan and 34 full-length isolates of PVX, Table S7: Details of nucleotide identities among TGB1 sequence of one PVX isolate from Pakistan and 34 full-length isolates of PVX. Table S8: Biological metadata of 219 full length CP nucleotide sequences of PVX, Table S9: Biological metadata of 219 full length CP amino acid sequences of PVX, Table S10: Details of pairwise identities among 632 (413 full length genomes + 219 others) CP nucleotide and amino acid sequences of PVX, Table S11: Details of existing PVX strains and approaches used in literature for their naming, Table S12: Details of virus isolates that have been assigned to proposed strain groups within species PVX, Table S13: Details of pairwise nucleotide identity among possible variants of strain PVX-A, Table S14: Details of pairwise nucleotide identity among possible variants of strain PVX-E, Table S15: Details of pairwise nucleotide identity among possible variants of strain PVX-H, Table S16: Details of pairwise nucleotide identity among possible variants of strain PVX-I, Table S17: Details of 50 recombinant isolates of PVX, Table S18: Genetic diversity within geographical populations of PVX, Table S19: Genetic differentiation among geographical populations of PVX, Table S20: Gene flow among geographical populations of PVX, Table S21: Genetic diversity within random populations of PVX, Table S22: Genetic differentiation among random populations of PVX, Table S23: Gene flow among random populations of PVX, Table S24: Genetic diversity within recombinant and non-recombinant genomes of PVX, Table S25: Genetic differentiation among recombinant and non-recombinant genomes of PVX, Table S26: Gene flow among recombinant and non-recombinant genomes of PVX. File S2: Document version of figures including, Figure S1: SDT pairwise identity distribution plot of CP nucleotide sequences of 413 full length PVX isolates, Figure S2: SDT pairwise identity distribution plot of CP amino acid sequences of 413 full length PVX isolates, Figure S3: SDT pairwise identity distribution plot of RdRp nucleotide sequences of 413 full length PVX isolates, Figure S4: SDT pairwise identity distribution plot of RdRp amino acid sequences of 413 full length PVX isolates, Figure S5: SDT pairwise identity distribution plot of TGB1 nucleotide sequences of 413 full length PVX isolates, Figure S6: SDT pairwise identity distribution plot of TGB1 amino acid sequences of 413 full length PVX isolates, Figure S7: SDT pairwise identity distribution plot of TGB2 nucleotide sequences of 413 full length PVX isolates, Figure S8: SDT pairwise identity distribution plot of TGB2 amino acid sequences of 413 full length PVX isolates, Figure S9: SDT pairwise identity distribution plot of TGB3 nucleotide sequences of 413 full length PVX isolates, Figure S10: SDT pairwise identity distribution plot of TGB3 amino acid sequences of 413 full length PVX isolates, Figure S11: SDT pairwise identity distribution plot of 632 full length CP nucleotide sequences of PVX, Figure S12: SDT pairwise identity distribution plot of 632 full length CP amino acid sequences of PVX, Figure S13: Maximum-likelihood (ML) method-based phylogenetic tree of PVX using available 413 full-length viral isolates. The evolutionary history was inferred using general time reversible (GTR) model implemented in MEGA X. Phylogenetic tree of PVX showing clustering of 17 old strains in genomic lineages of PVX-A (14), PVX-E (2) and PVX-G (2), Figure S14: Comparison among previous and current recombination analyses of PVX using Recombination Detection Program (RDP), including the number of recombinants, detected recombination events, breakpoint patterns, recombinant fragment length, previously reported recombination positions, and newly identified crossover sites, Figure S15: Best fit model plot generated by Genetic Algorithm for Recombination Detection (GARD) using 413 full length genomes of PVX. Left: the best placement of breakpoints inferred by the algorithm for each number of breakpoints considered. Right: the improvement in the c-AIC score between successive breakpoint numbers (log scale), Figure S16: Total phylogenetic tree length by partition constructed using individual genomic fragments of 413 full length PVX sequences from which recombination signals were detected by Genetic Algorithm for Recombination Detection (GARD), Figure S17: Natural host range of potato virus X, Figure S18: Diagnostic host range of potato virus X.
