# Supplementary material for: Global Genetic Diversity, Provisional Strain Demarcation, and Evolutionary Dynamics of Potato Virus X (PVX)
Source: Viruses. 2026 Sep 2;18(9):968. doi: 10.3390/v18090968 (PMC13611960; doi:10.3390/v18090968)
Supplement: Supplementary file 1 [file viruses-18-00968-s001.zip › Supplementary File S2.pdf]

(1)

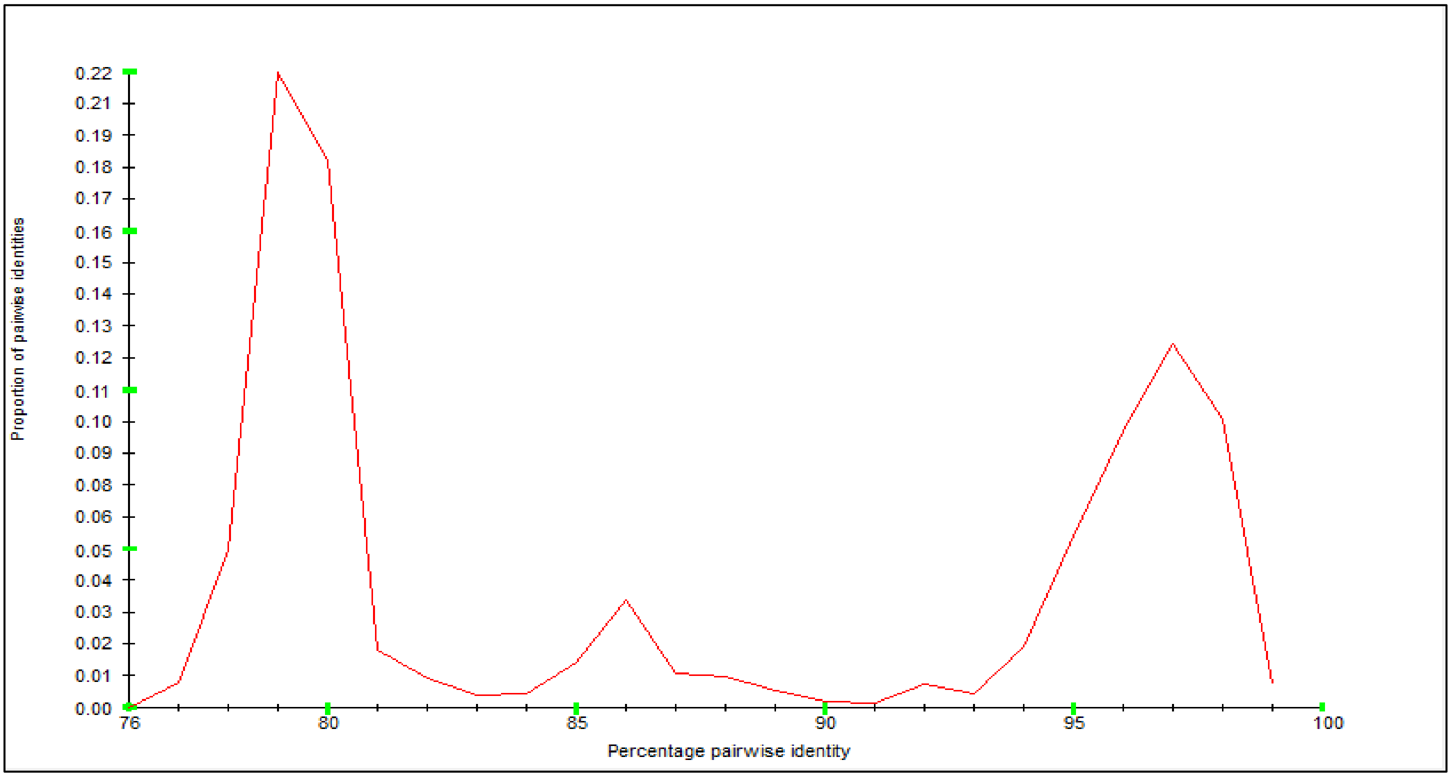

Figure S1: SDT pairwise identity distribution plot of CP nucleotide sequences of 413 full length PVX isolates

(2)

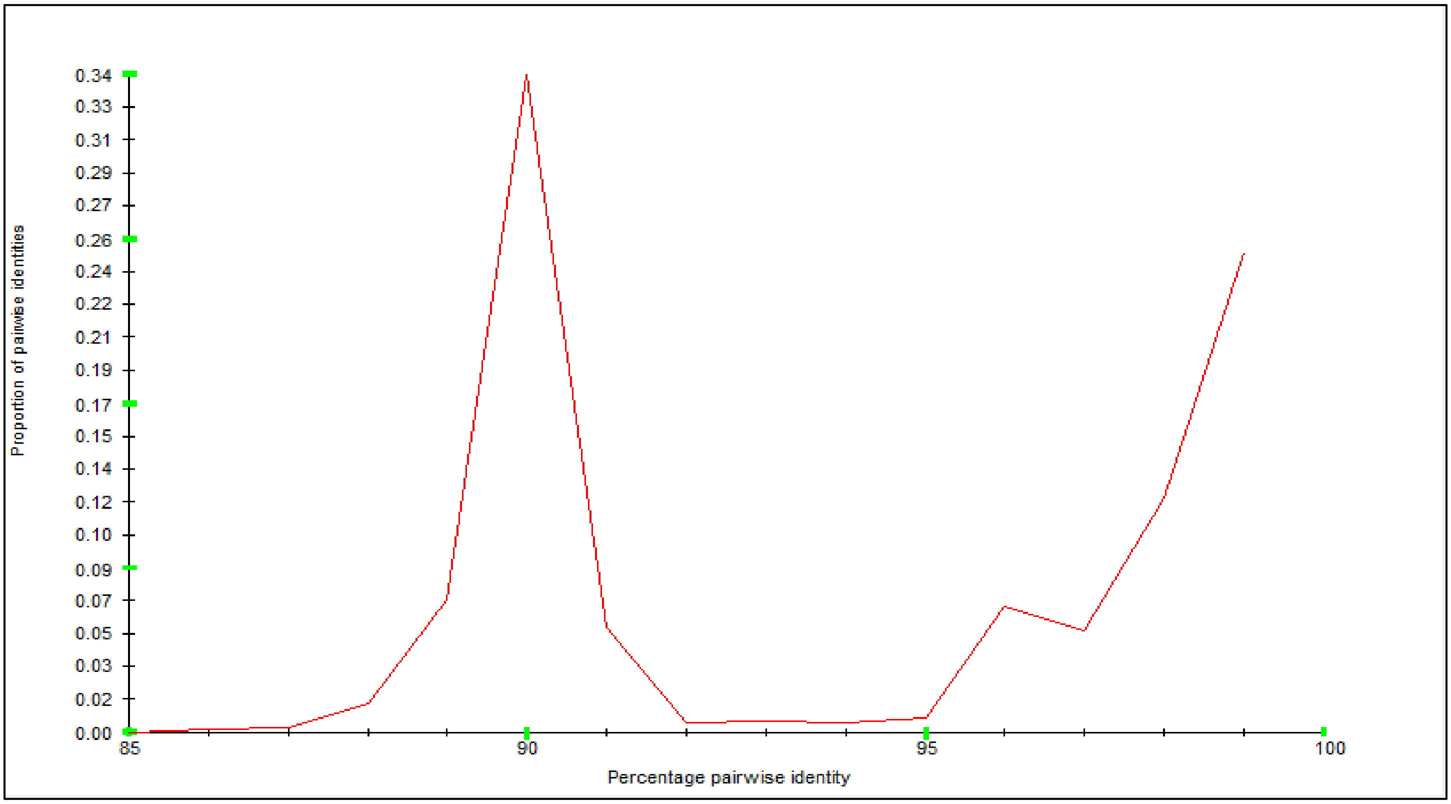

Figure S2: SDT pairwise identity distribution plot of CP amino acid sequences of 413 full length PVX isolates

(3)

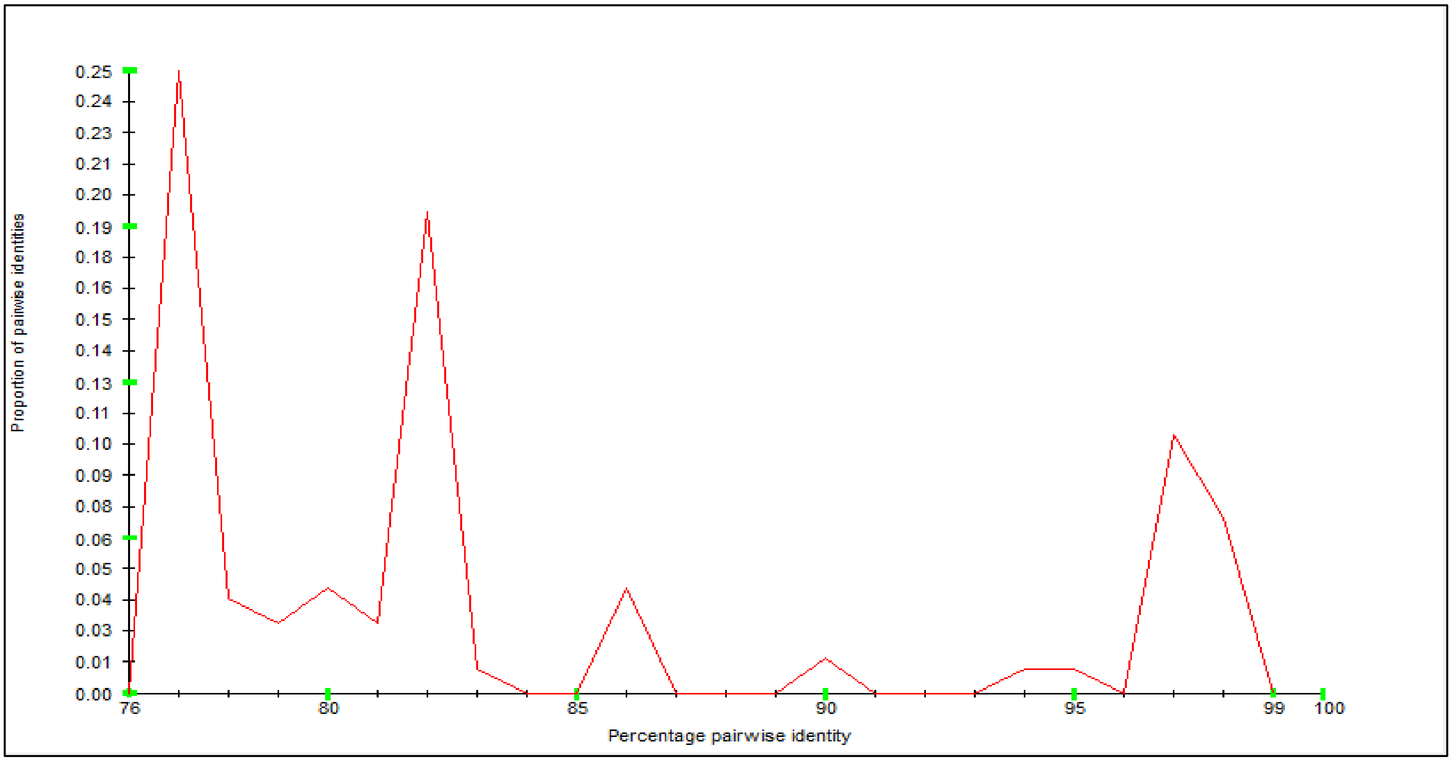

Figure S3: SDT pairwise identity distribution plot of RdRp nucleotide sequences of 413 full length PVX isolates

(4)

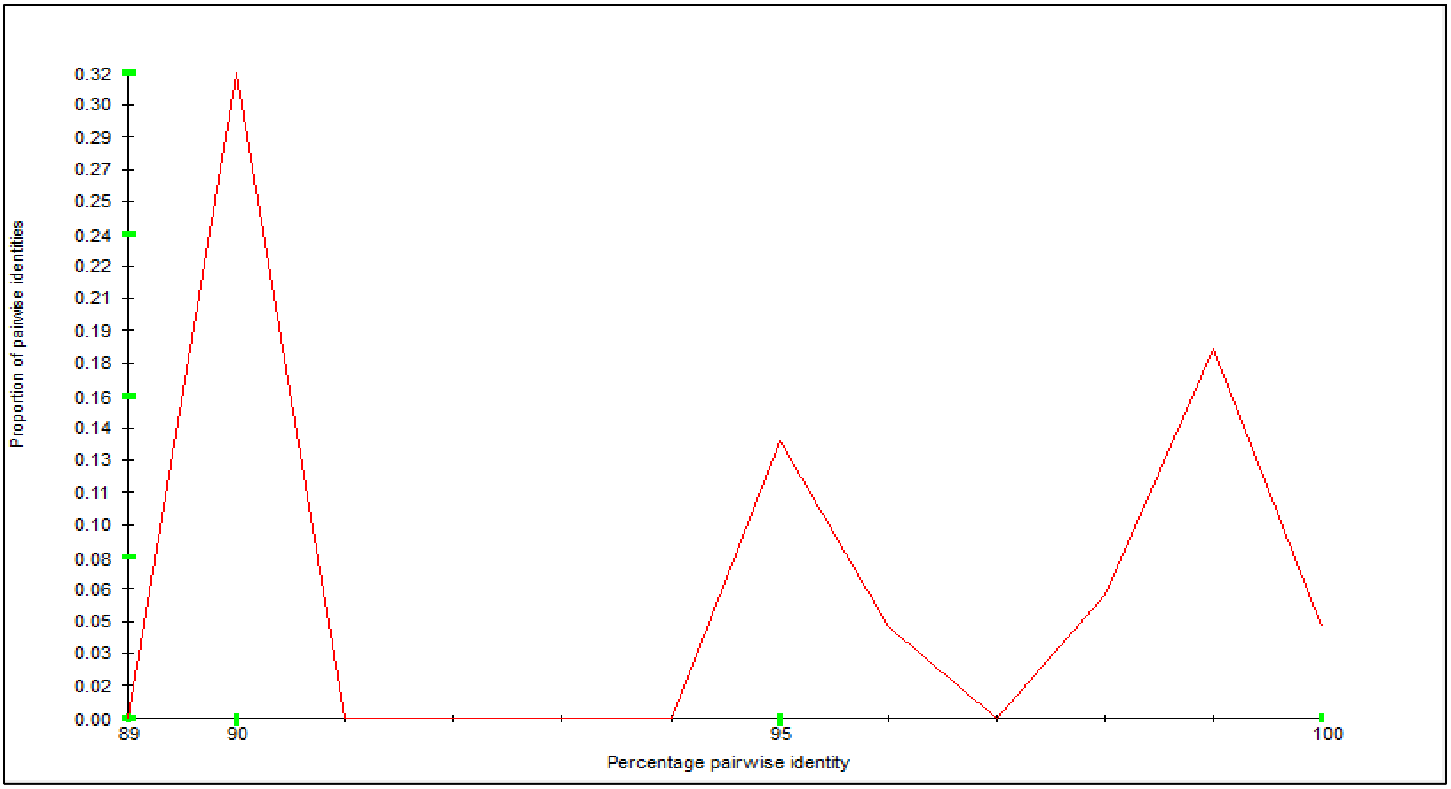

Figure S4: SDT pairwise identity distribution plot of RdRp amino acid sequences of 413 full length PVX isolates

(5)

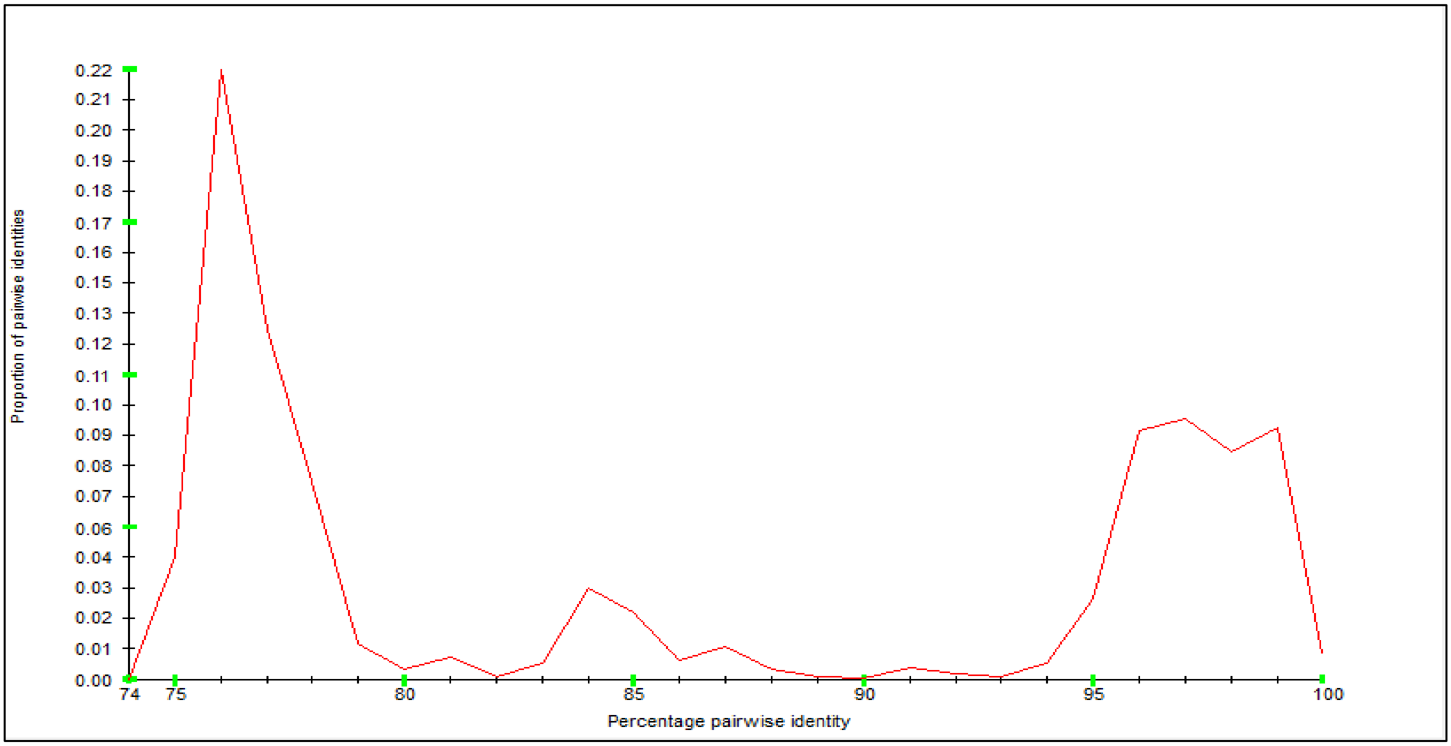

Figure S5: SDT pairwise identity distribution plot of TGB1 nucleotide sequences of 413 full length PVX isolates

(6)

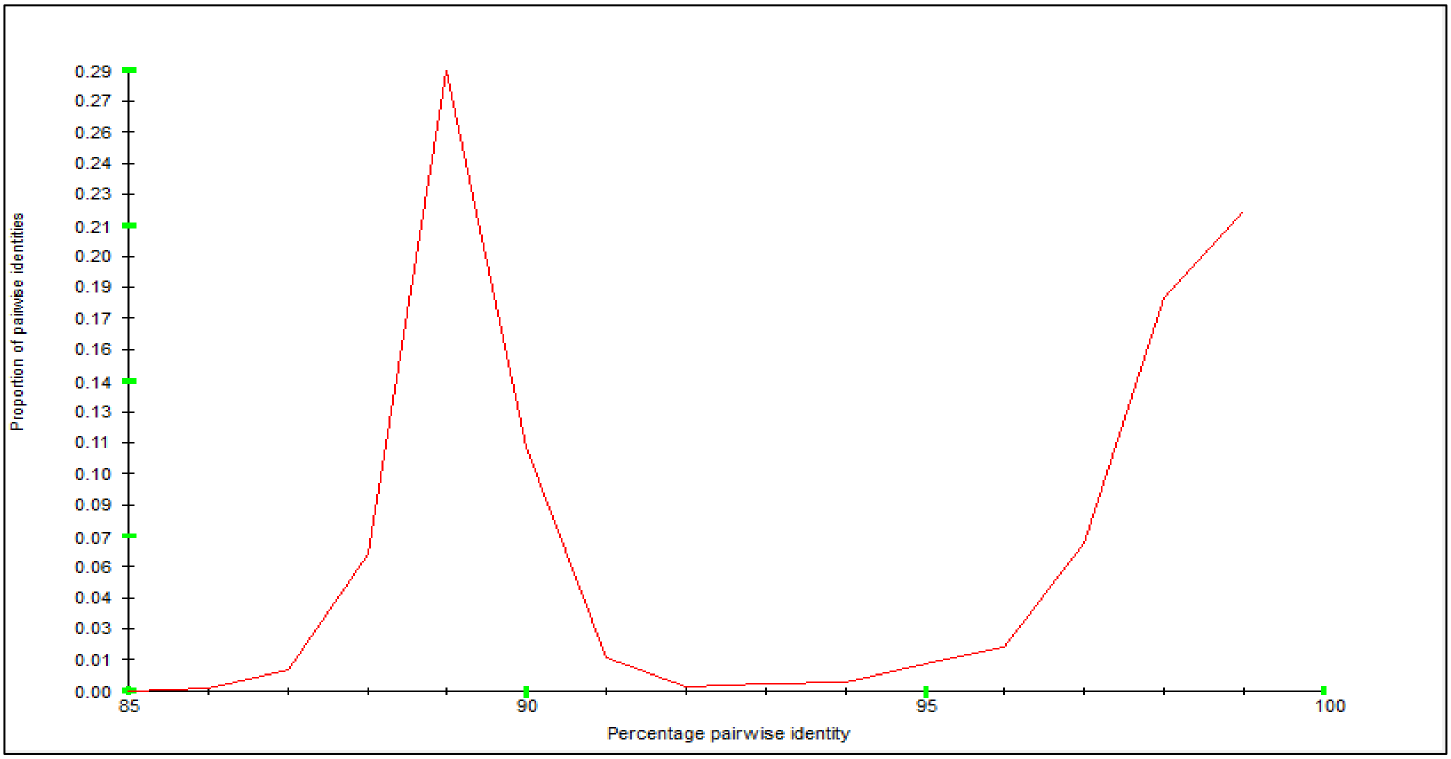

Figure S6: SDT pairwise identity distribution plot of TGB1 amino acid sequences of 413 full length PVX isolates

(7)

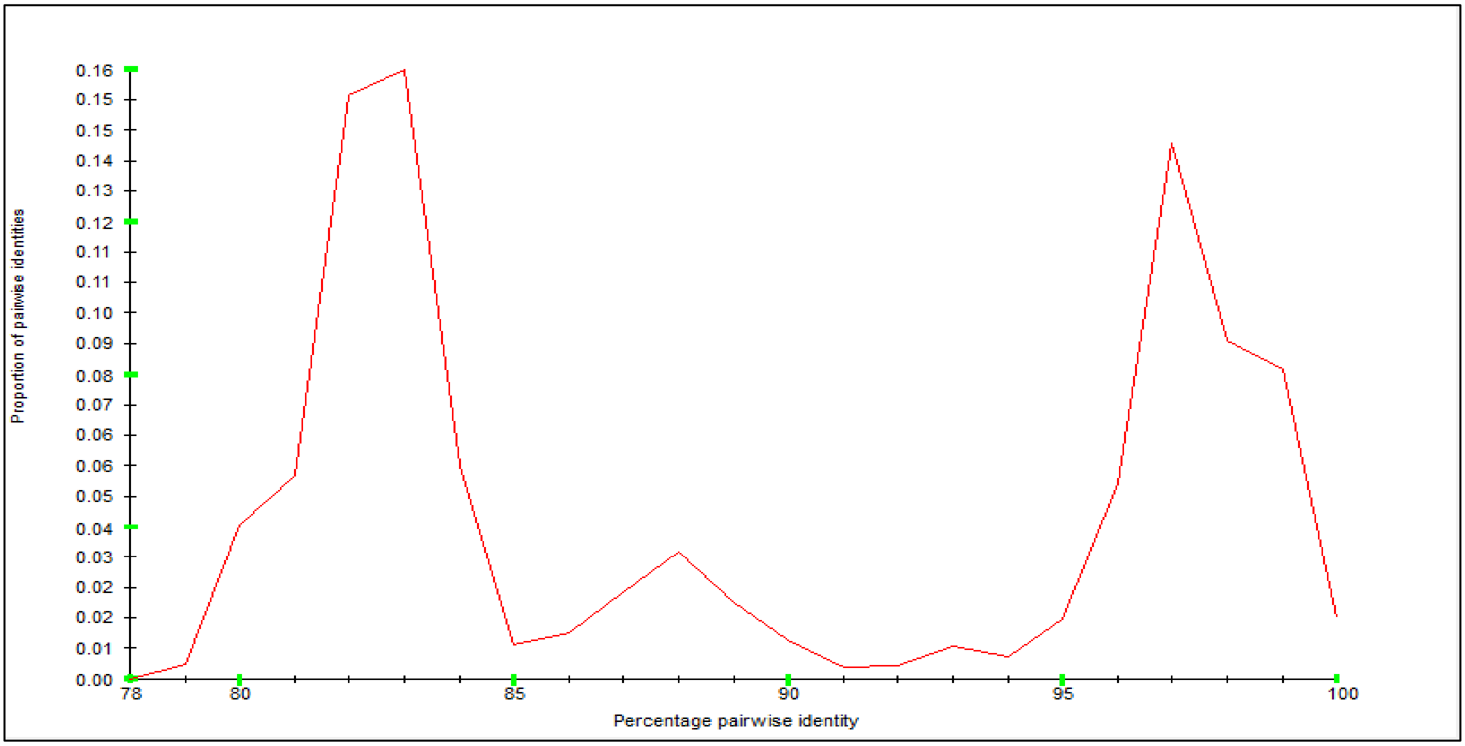

Figure S7: SDT pairwise identity distribution plot of TGB2 nucleotide sequences of 413 full length PVX isolates

(8)

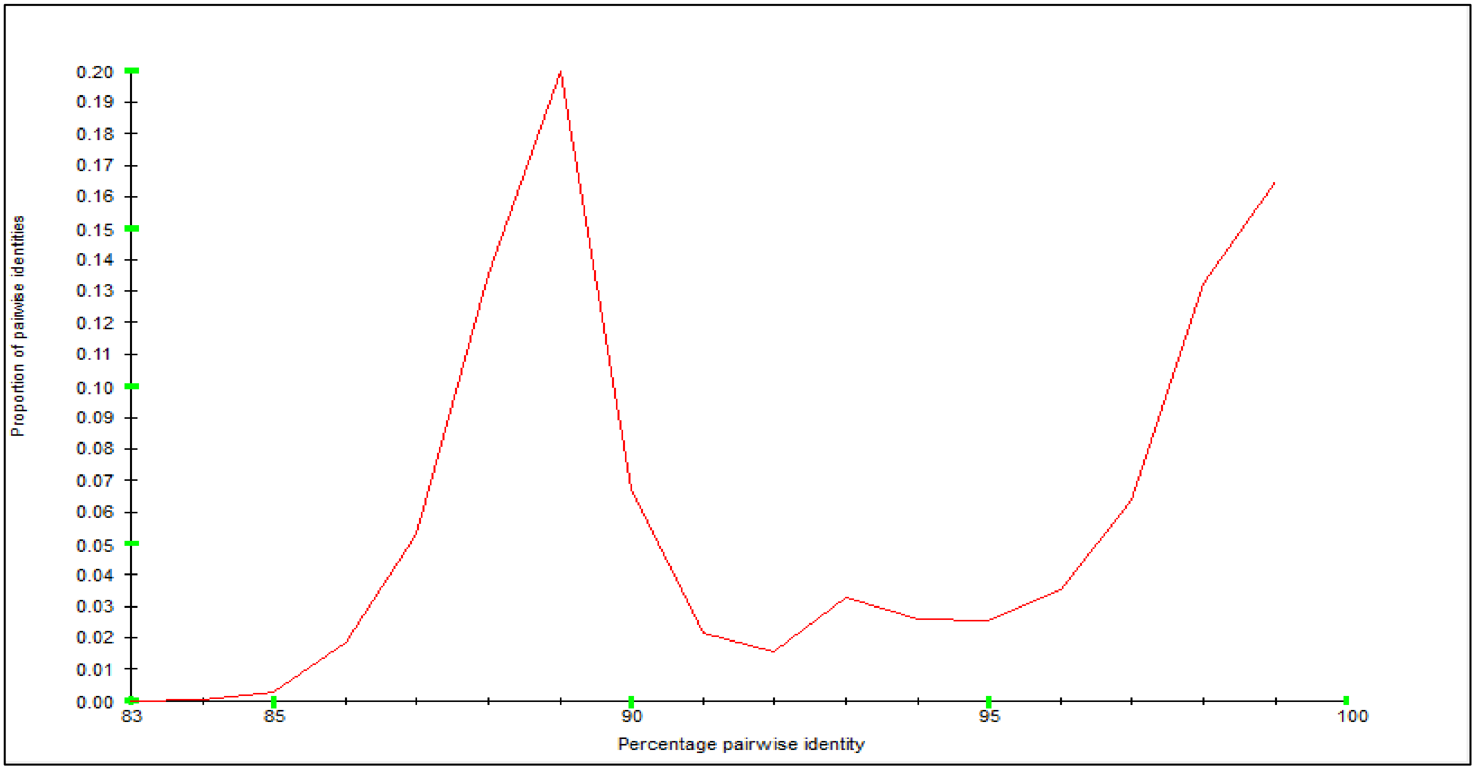

Figure S8: SDT pairwise identity distribution plot of TGB2 amino acid sequences of 413 full length PVX isolates

(9)

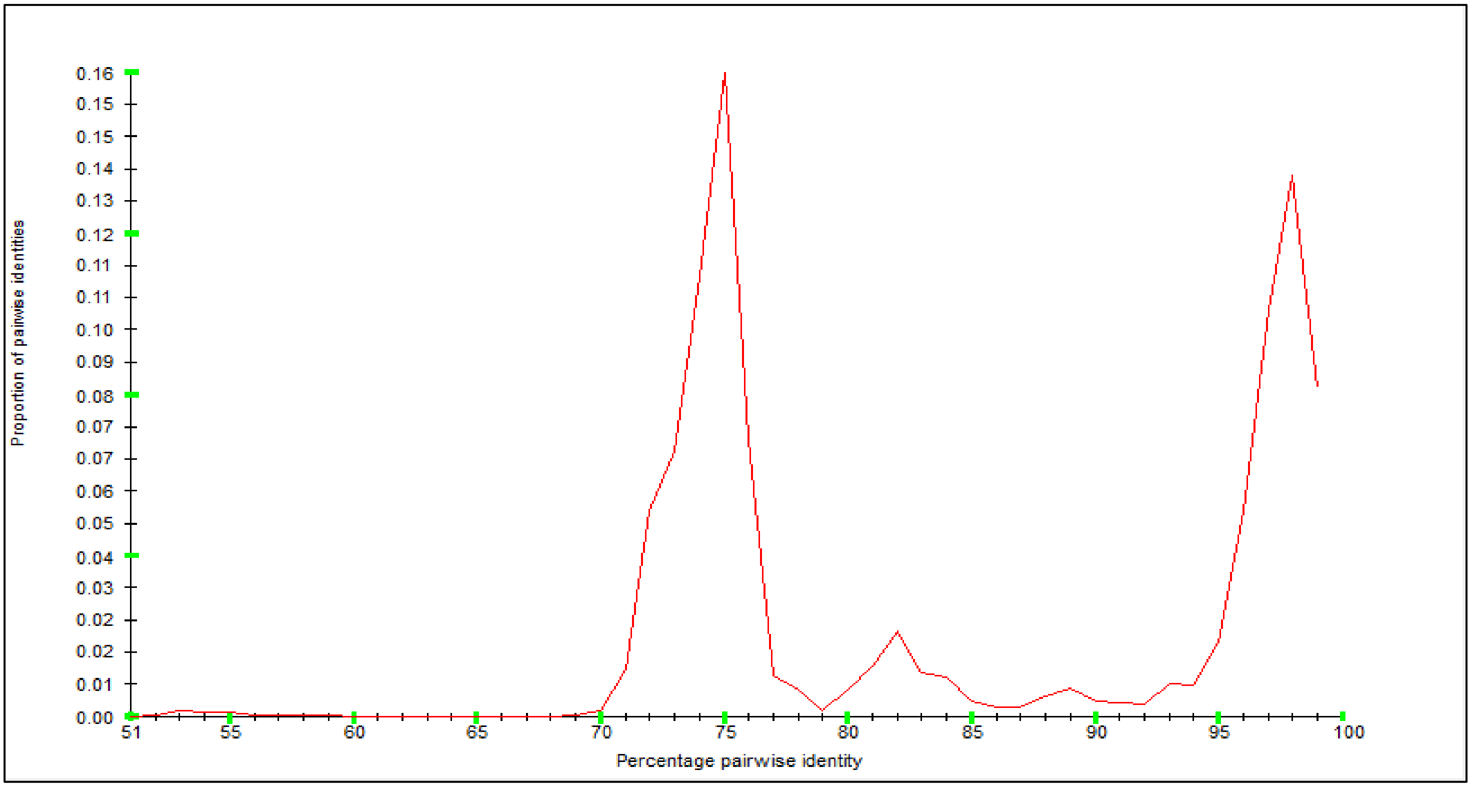

Figure S9: SDT pairwise identity distribution plot of TGB3 nucleotide sequences of 413 full length PVX isolates

(10)

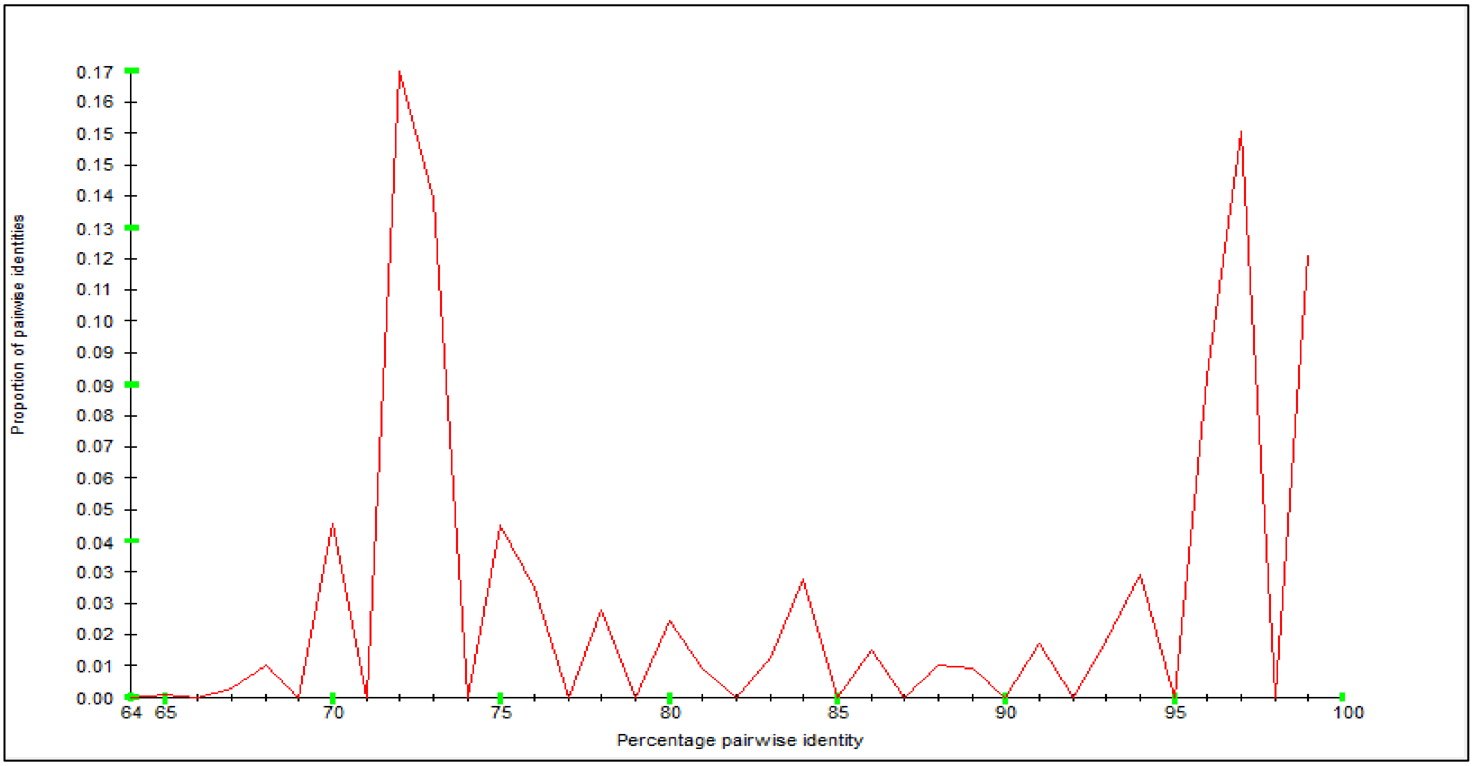

Figure S10: SDT pairwise identity distribution plot of TGB3 amino acid sequences of 413 full length PVX isolates

(11)

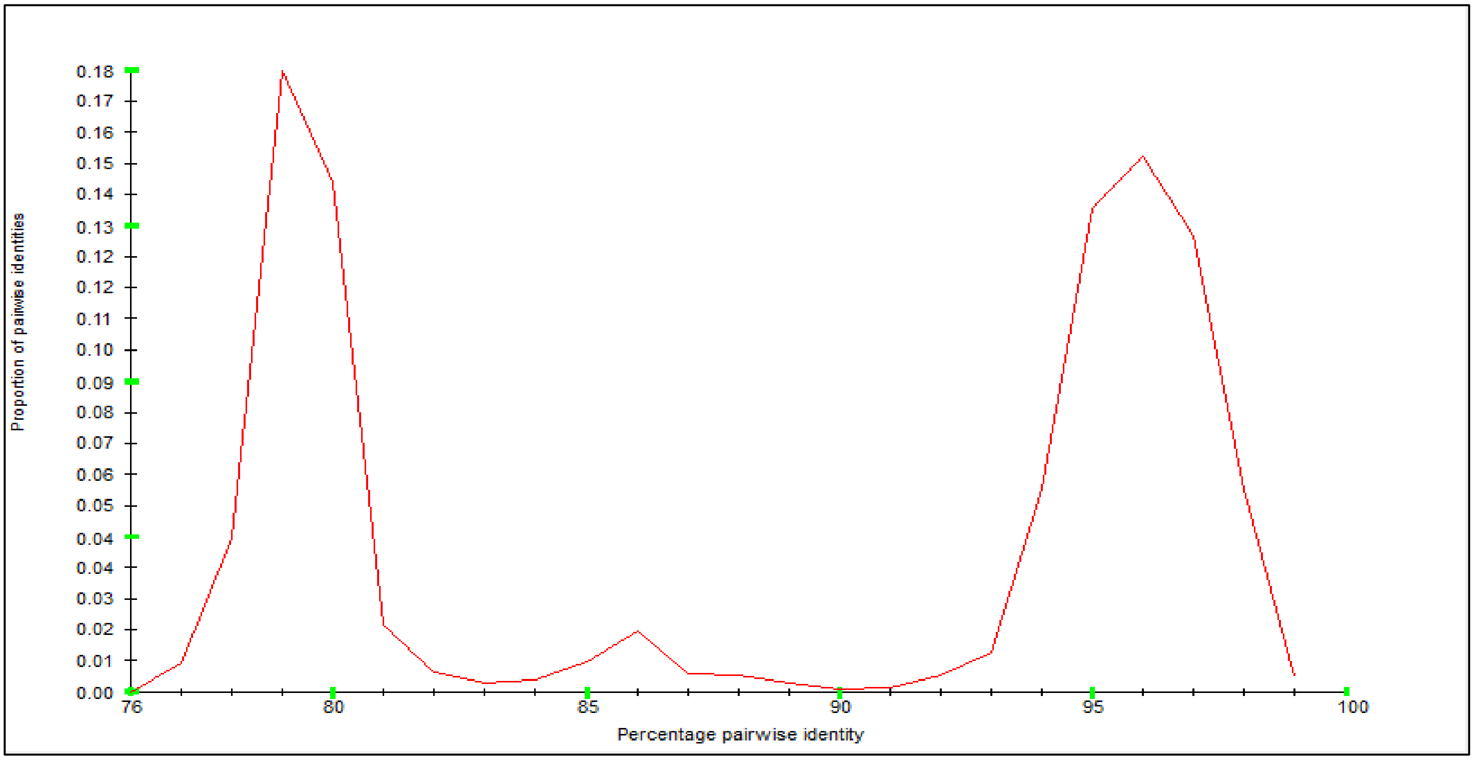

Figure S11: SDT pairwise identity distribution plot of 632 full length CP nucleotide sequences of PVX

(12)

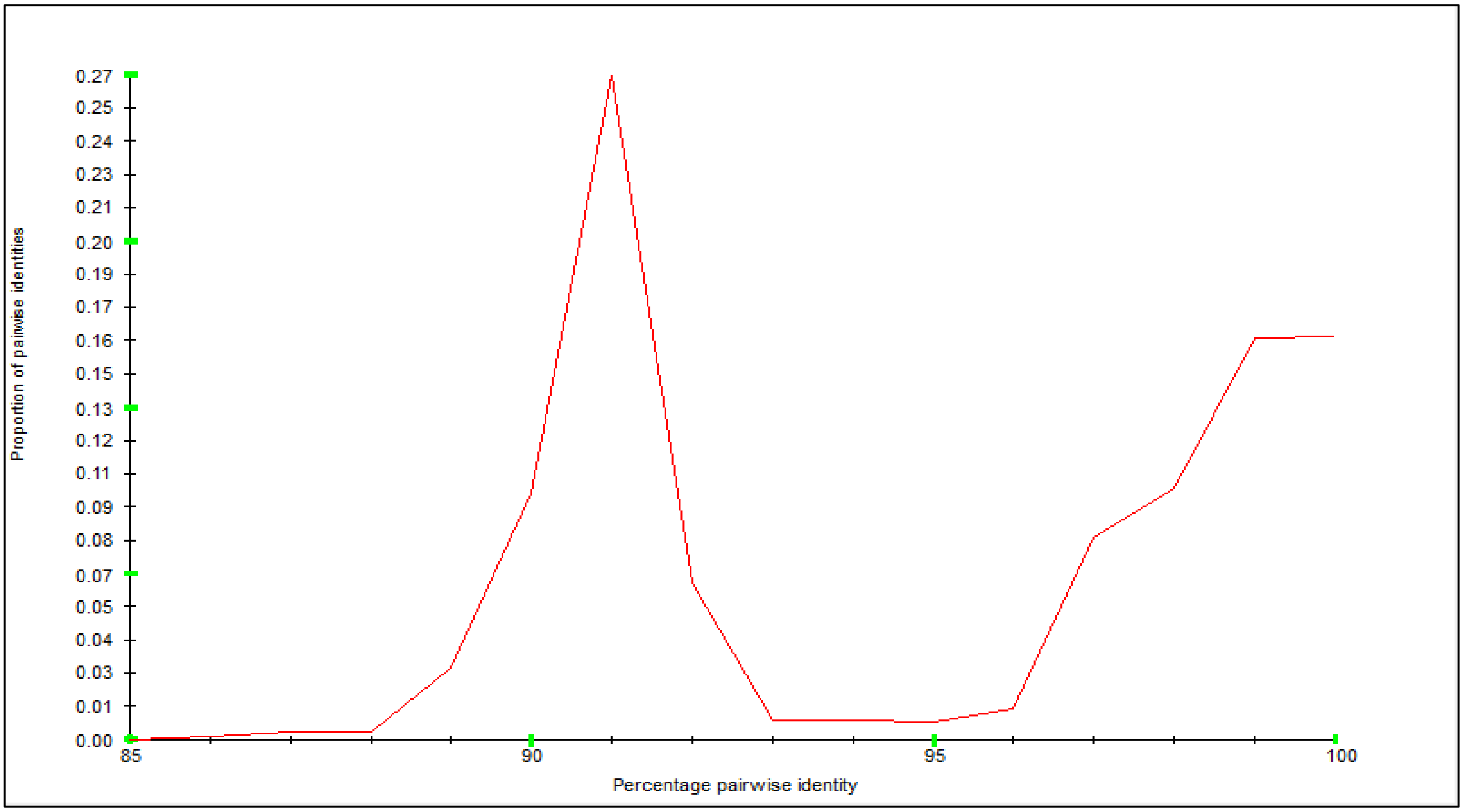

Figure S12: SDT pairwise identity distribution plot of 632 full length CP amino acid sequences of PVX

(13)

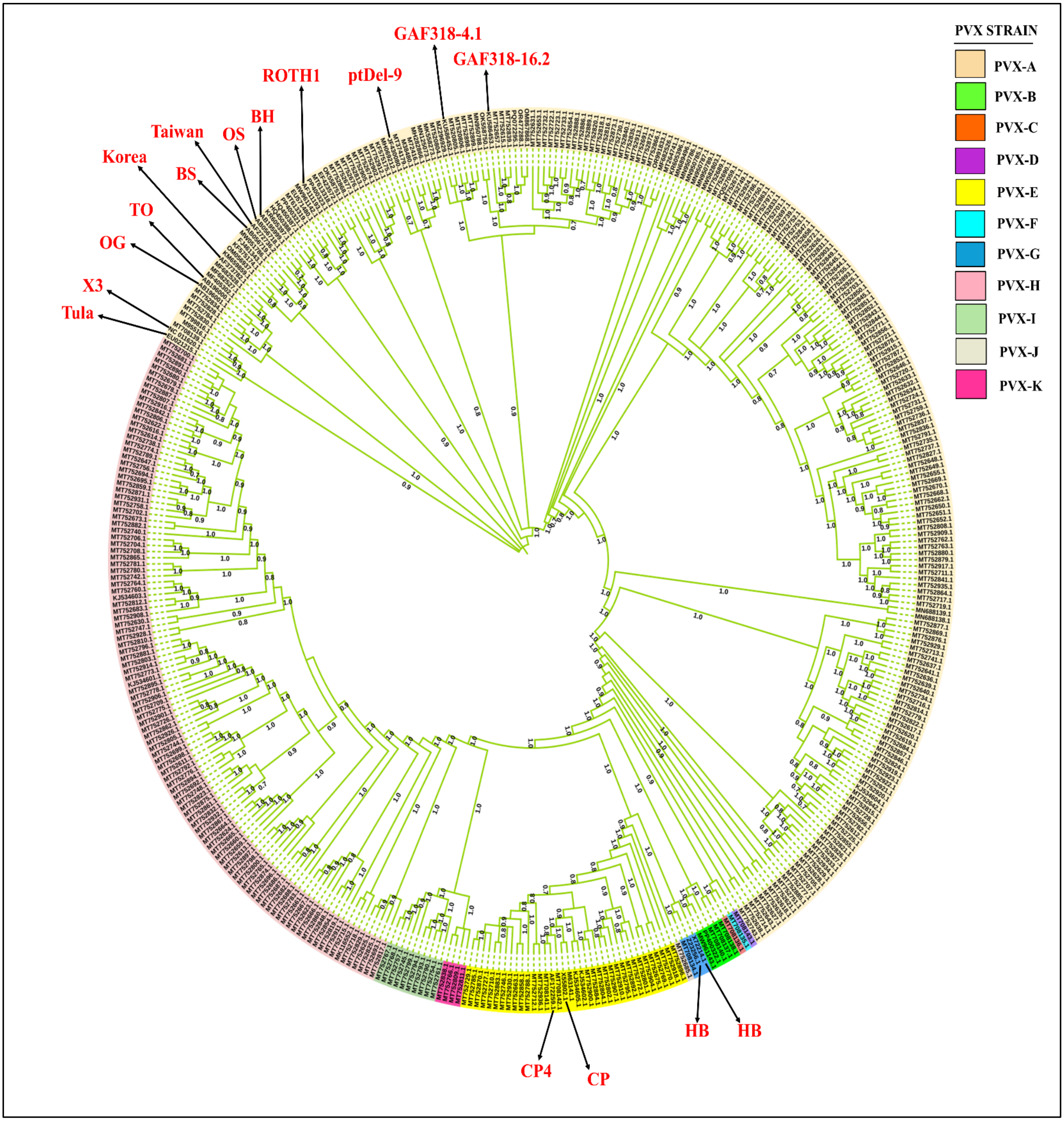

Figure S13: Maximum-Likelihood (ML) method based phylogenetic tree of PVX using available 413 full-length viral isolates. The evolutionary history was inferred using General Time Reversible (GTR) nucleotide model implemented in MEGA X. Phylogenetic tree of PVX isolates showing clustering of 17 old strains in genomic lineages of PVX-A (13), PVX-E (2) and PVX-G (2).

(14)

Previous analysis **Updated analysis**

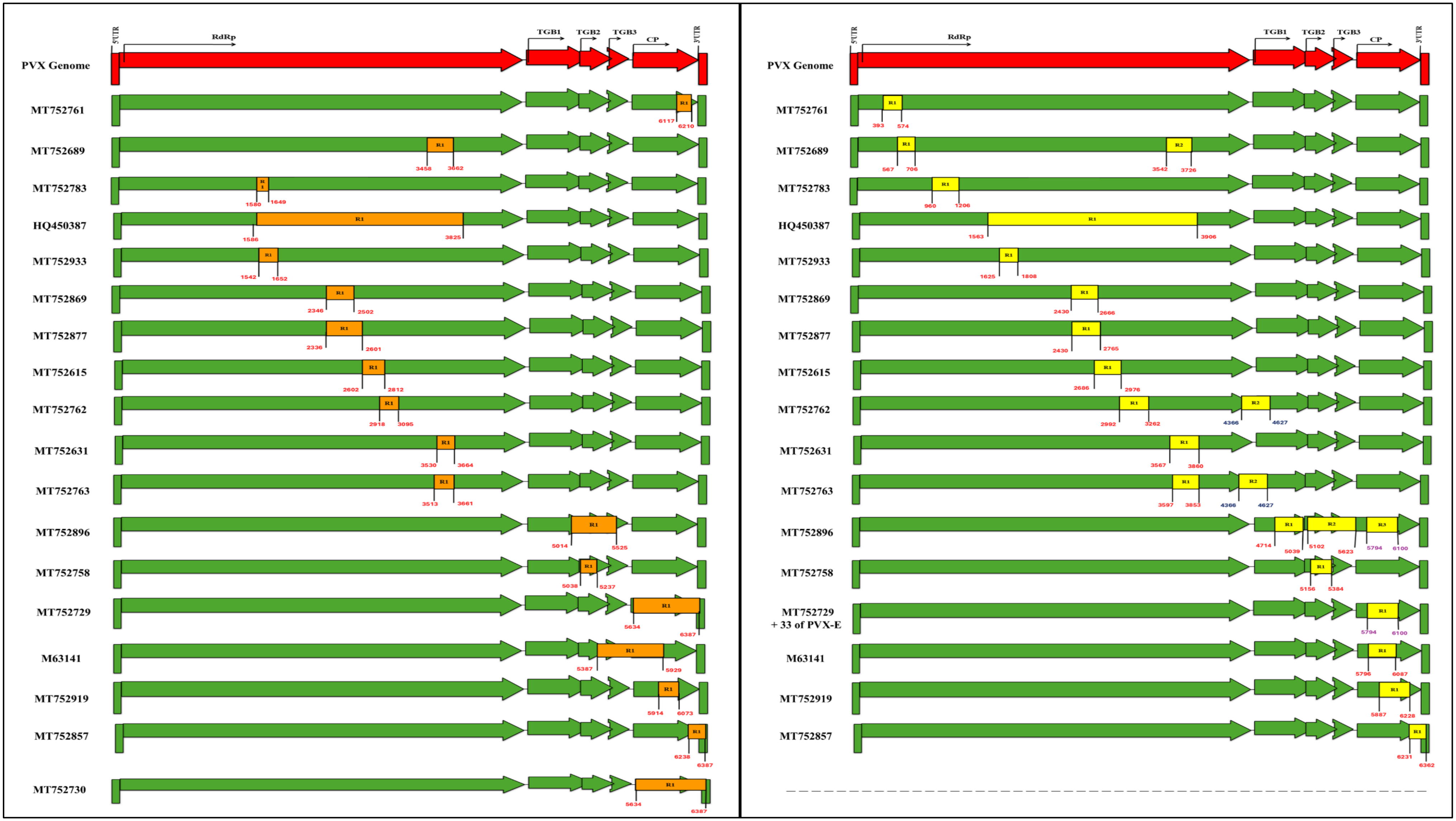

Figure S14: Comparison among previous and current recombination analyses of PVX using Recombination Detection Program (RDP), including the number of recombinants, detected recombination events, breakpoint patterns, recombinant fragment length, previously reported recombination positions, and newly identified crossover sites.

(15)

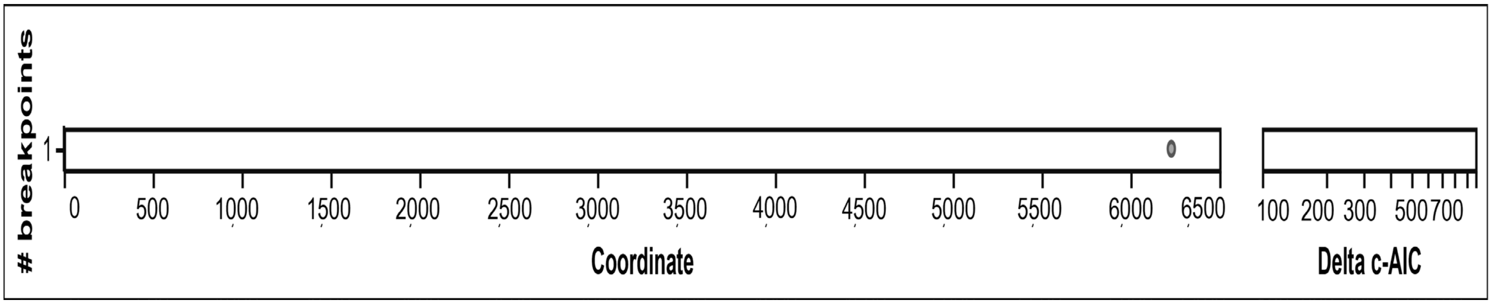

Figure S15: Best fit model plot generated by Genetic Algorithm for Recombination Detection (GARD) using 413 full length genomes of PVX. **Left**: the best placement of breakpoints inferred by algorithm for each number of breakpoints considered. **Right**: the improvement in the c-AIC score between successive breakpoint numbers (log scale) sites.

(16)

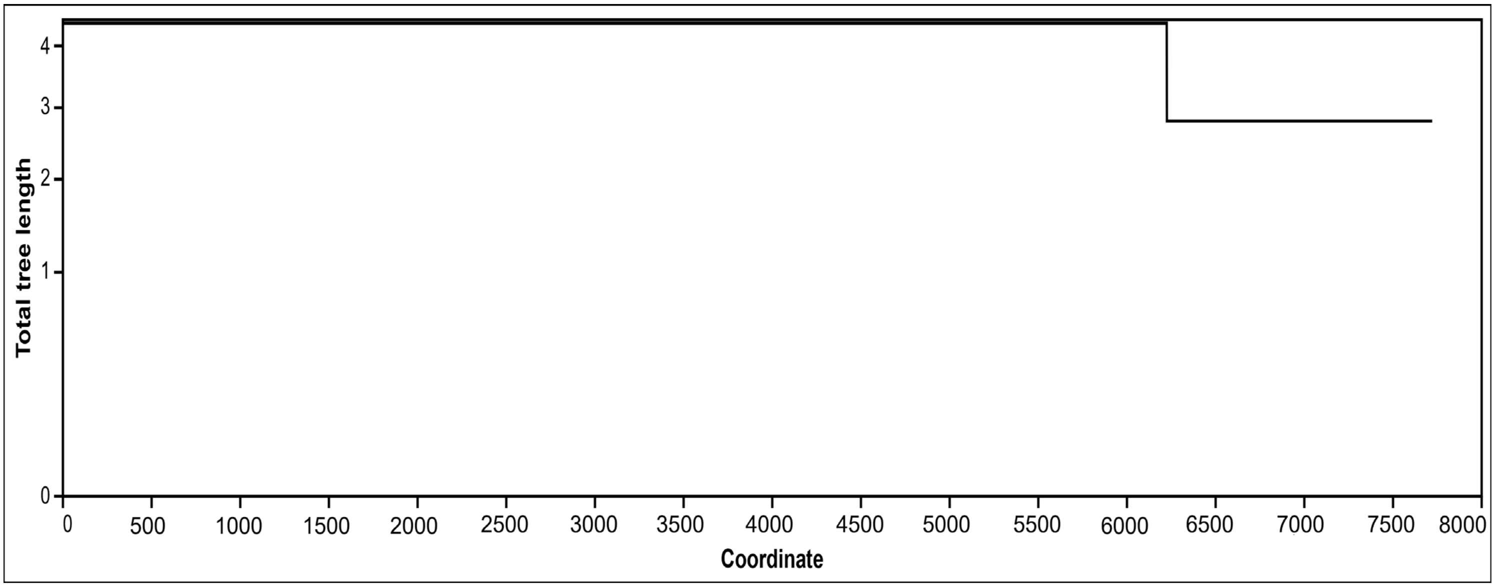

Figure S16: Total phylogenetic tree length by partition constructed using individual genomic fragments of 413 full length PVX sequences from which recombination signals were detected by Genetic Algorithm for Recombination Detection (GARD)**.**

(17)

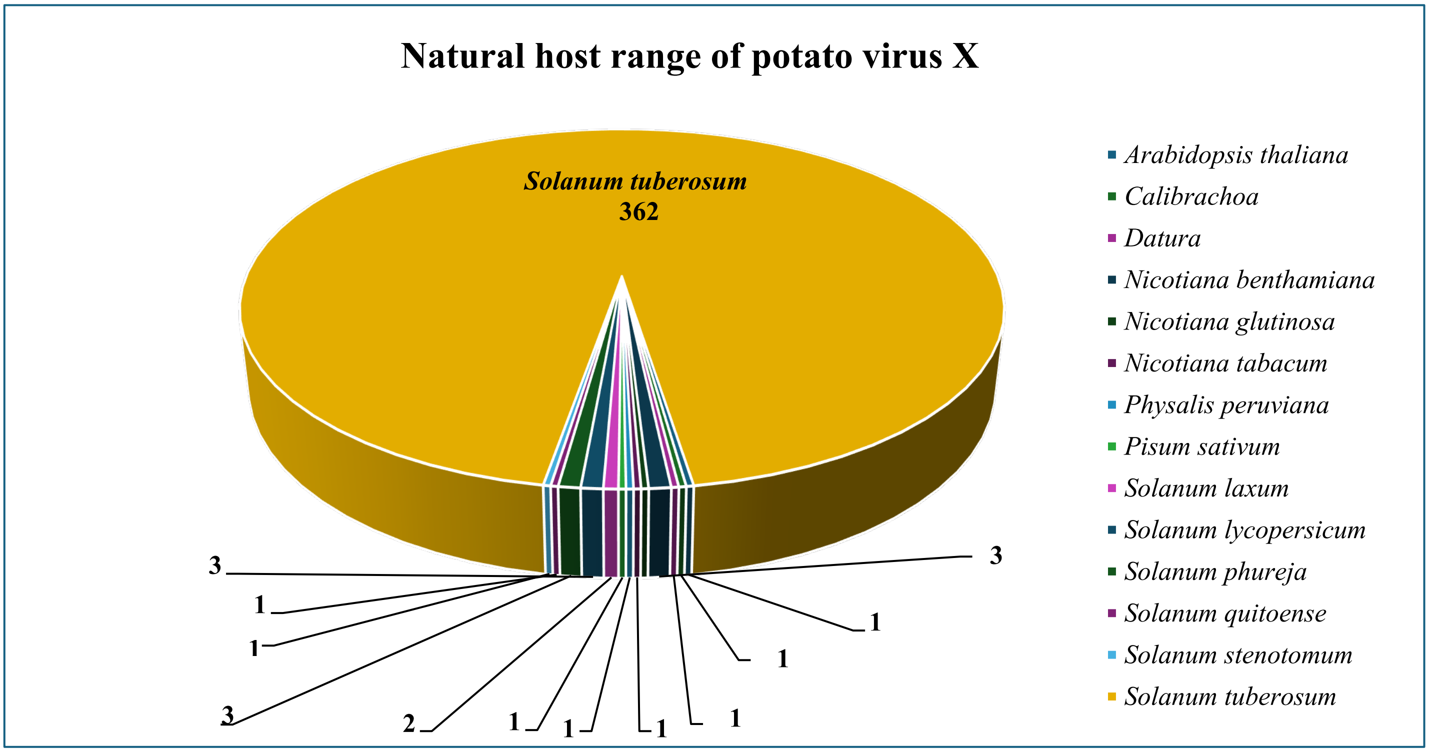

Figure S17: Natural host range of potato virus X

(18)

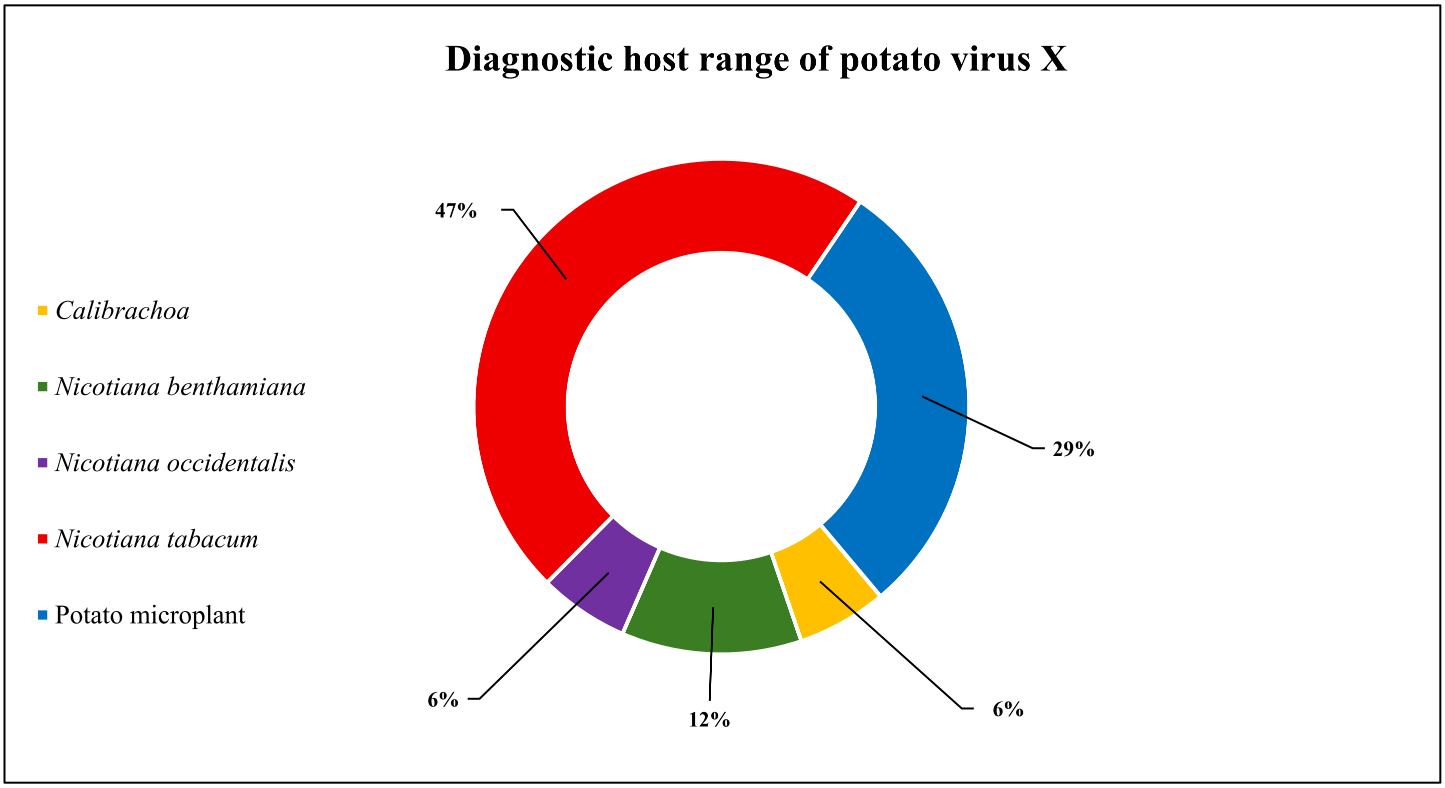

Figure S18: Diagnostic host range of potato virus X
